# A New Avrami-Based Exponential Model for Predicting Fiber-Reinforced Polymer Bar Service Life: A Comparison with Existing Models Using a Large Database

**DOI:** 10.3390/polym16212956

**Published:** 2024-10-22

**Authors:** Tuanjie Wang, Abdul Ghani Razaqpur, Shaoliang Chen, Shiqiang Zhou

**Affiliations:** 1 Henan Engineering Technology Research Center of Industrial Waste Gypsum Application, School of Architecture and Civil Engineering, Zhengzhou University of Industrial Technology, Zhengzhou 451150, China; wangtuanjie@zzuit.edu.cn; 2 Department of Civil Engineering, McMaster University, Hamilton, ON L8S 4L7, Canada; 3 State Key Laboratory of Green Building Materials, China Building Materials Academy Co., Ltd., Beijing 100024, China; chenshaoliang@mail.nankai.edu.cn; 4 Zhengzhou Research Institute, Beijing Institute of Technology, Zhengzhou 450000, China; shiqiang.zhou@bit.edu.cn

**Keywords:** Avrami equation, database, FRP bar, prediction model, service life, tensile strength retention

## Abstract

The fiber-reinforced polymers (FRP) bar is a promising solution to problems caused by steel rebar corrosion in concrete. To assess the service life of the FRP bar based on accelerated test results, it is crucial to have a reliable model. Here, a modified exponential (MEP) model is proposed based on the Avrami equation. The Avrami equation provides a theoretical foundation for the empirical exponential (EP) model and does not a priori fix the power of the exposure time to one. A database containing 903 data points from 74 groups of test specimens is assembled to compare the reliability of the MEP model vis-a-vis the EP, single logarithmic, double logarithmic, and power function models. The combination of Root Mean Square Error (RMSE), the Mean Absolute Error (MAE), and the coefficient of determination (*R*^2^) criteria is proposed for assessing model reliability. It is shown that in certain cases the combined criteria, versus *R*^2^ alone, significantly increase the number of test groups meeting the acceptable performance limit. Observed test data aberrations are found to have minor influence on the results of the EP model, but they significantly influence the results of the other four models. The EP model generally predicts the lowest activation energy and the smallest strength retention for similar groups of bars, while the predicted values of the other four models exhibit a relatively small difference. The difference between the predicted strength retention values of the EP and MEP models shows an increasing trend with the increase of the absolute value of (1 − *n*), where *n* is the power of the exposure time in the MEP model.

## 1. Introduction

### 1.1. The Current State, Purpose and Significance

Steel reinforced concrete is the most prevalent construction material in modern infrastructure. However, the corrosion of steel rebars within concrete structures exposed to aggressive agents, including seawater, marine vapor, and deicing salts, poses a significant problem. Rectifying this issue is costly, while neglecting it can be dangerous. One promising solution to combat the problem of steel corrosion in concrete is the replacement of steel rebars by fiber-reinforced polymer (FRP) bars [1]. But FRP bars are also susceptible to degradation when exposed to aggressive environmental conditions. These conditions may include elevated temperatures, high humidity, alkaline and acidic solutions, freeze–thaw cycling, wet–dry cycling, ultraviolet radiation, and their combinations [2]. Consequently, the widespread adoption of FRP bars necessitates understanding their long-term strength retention under realistic service conditions. This understanding can be achieved through either direct observation of FRP bar performance in structures over extended periods, typically spanning decades, or through extrapolation of their performance in accelerated tests under laboratory conditions. The former is not practical, due to the relatively recent introduction of FRP bars as concrete reinforcement and the lengthy duration required for gathering long-term field data. Furthermore, it is difficult to control the elements under field conditions. Even though durability of GFRP bars embedded in some actual concrete bridges and harbor structures was evaluated after exposure to the natural environment for 15 to 20 years [3,4,5], these data cannot be used to check the reliability of a model due to the lack of enough data. In contrast, accelerated tests are characterized by much a shorter duration and a precisely controlled test environment. Consequently, the latter has become the method of choice [2,6].

To extrapolate accelerated test results to long-term performance, a mathematical model needs to be adopted. The basic models involve establishment of a relationship between the bar’s retained tensile strength and exposure time based on accelerated tests, supplemented by the utilization of a so-called time shift factor (TSF) [7]. Each model has some parameters which must be calibrated using accelerated test results. The exponential (EP), single logarithmic (SL), double logarithmic (DL), and power function (PF) models are commonly adopted [6,8,9,10]. They contain the exposure time as a parameter, but not the exposure temperature. Temperature dependence is checked through the Arrhenius equation. Furthermore, to consider the fluctuations in temperature and humidity, as well as factors such as concrete cover and service life, refinements have been introduced through the time shift factor (TSF) [11,12,13]. More recently, the writers in [14] proposed a new model based on the Avrami–Erofe’ev Equation [15] and Eyring’s Transition State Theory, which obviates the use of TSF [7].

The second type of basic model is based on moisture diffusion, where strength retention is related to either the bar mass change [16] or its diffusion coefficient [17]. Initially, models were based on the assumption that at a given time, all parts of the bar cross-section permeated by the immersing solution will completely lose their strength [18]. Subsequently, refinements were introduced by assuming partial loss of strength due to solution permeation [19]. But, as indicated by Bhise [17], strength-retention estimation based on Fickian diffusion law may only be suitable for analyzing results of short-term tests while Langmuir’s diffusion law may be more appropriate for predicting long-term strength loss. On the other hand, determination of the four parameters of the latter law can be onerous [20].

In addition to the basic models, data-driven models for GFRP bars have been proposed, including those based on optimized tree-based random forest [21], fuzzy metaheuristic [22], non-linear genetic [23], and computational AI [24]. These models go beyond environmental parameters such as temperature, pH, and exposure time, allowing for the analysis of the effects of bar size and fiber volume fraction on bar durability. This approach has also been applied to study the effect of confinement by FRP reinforcement on concrete column strength [25,26].

Among the foregoing models, Type I basic models, such as EP, SL, DL, and PF are widely applied by researchers [6,27]. To assess their reliability, the coefficient of determination (*R*^2^) is used as the measure of the goodness-of-fit to the companion experimental data. Generally, the minimum *R*^2^ value for an acceptable model is considered to be 0.80 [28,29]. Note that each line corresponding to different accelerated temperatures will have its own *R*^2^ value, which may differ to the point that some may not meet the minimum acceptability criterion. This situation could make the acceptance and/or rejection of the model based on the *R*^2^ value alone problematic.

While *R*^2^ can measure the reliability or goodness-of-fit of the model, it cannot measure its bias or accuracy. Assessment of bias is particularly crucial for models that are applied well beyond the range of the independent variables used in the experiments. Since FRP accelerated tests are conducted for relatively short time periods, with time being the independent variable, extension of their results to long service lives spanning decades could be fraught with difficulties. The difficulties may be partially addressed by supplementing *R*^2^ with other metrics to measure a model’s accuracy and reliability.

In addition, it is common practice [7,8,9,29,30,31,32,33,34,35,36] to check whether the Type I models obey the Arrhenius equation. The check is performed by plotting the data corresponding to different percentages of tensile strength retention in an Arrhenius diagram. The coordinate system in the diagram comprises 1/*T* as the abscissa and ln(*t*) as the ordinate, where T represents the exposure temperature and ln(*t*) the natural logarithm of the time needed to reach a specified tensile strength retention (TSR). Next, to each set of data corresponding to a specified TSR, a straight line is fitted. If the fitted lines appear parallel, the model is assumed to obey the Arrhenius equation and is deemed satisfactory [8,9,10]. The parallelism of the lines means they have the same slope. The slope represents the *E_a_/R* of the FRP bar under investigation. The quantity *E_a_* is the activation energy of the chemical reaction causing degradation of the bar and *R* is the gas constant. However, this check neither proves nor disproves the accuracy of the strength–time relationship in a model.

For the SL and DL models, the absence of parallelism was reported by some researchers [8,9,10,17,32]. If strength retention is assumed to be zero after infinite exposure time, the EP model has been reported to always [9,30,31,34,35,36] exhibit perfectly parallel lines in the Arrhenius diagram. An important question is the following: what is the key parameter influencing parallelism of the fitted lines in these models? It is currently common to judge parallelism visually [7,8,9,29,30,31,32,33,34,35,36], but this is subjective. There is need for an objective mathematical criterion to ascertain the existence of parallelism. The present research work addresses these issues and furnishes some reasonable solutions. Finally, to date, comparison among the results of the above models has been based on a relatively small number of test data [6,8,10]. It has been reported [6,8,10] that the exponential model or its refined form is the most reliable degradation model. To the best of the writers’ knowledge, this conclusion is not validated by a sufficiently large and robust database. Accordingly, in this study, a database comprising 74 data groups (903 data points), reported in 29 publications from 2008 to 2024, is established, and it is used to check the accuracy and reliability of the above models. It is shown that compared to the SL, DL and PF models, the EP model is very sensitive to differences among the observed retained strength values under different exposure temperatures in accelerated tests. This sensitivity results in a high rejection rate of the bars, due to insufficient durability. It is shown here that the high rejection rate is a consequence of the deficiency of the model. Consequently, a modified exponential model (MEP) is proposed to eliminate this deficiency. The reliability and accuracy of the proposed model is compared with those of the existing models using well-known statistical measures. The outcome is that the proposed model is reliable and is underpinned by a well-known theory of materials degradation kinetics, known as Avrami’s equation [15].

### 1.2. Review of Traditional FRP Service-Life Prediction Models

As described below, in existing models, generally the bar retained tensile strength is expressed as a function of the exposure time and temperature via separate functions.

#### 1.2.1. Temperature Dependency

Svante Arrhenius [37] initially proposed Equation (1) for the chemical kinetics of gases.

(1) $$k=A\exp(-\frac{E_{a}}{RT})$$

where *k* is the rate constant, *A* is the frequency factor or the pre-exponential, *E_a_* is the activation energy (kJ·mo1^−1^), *R* is the gas constant (8.314462 J/K·mol), and *T* is the absolute temperature. The frequency factor *A* is generally assumed to be temperature-independent for a limited temperature range (≤50 K) [37]. 

The earliest application of Equation (1) to construction materials is attributed to Litherland et al. [38], who used it to model glass-fiber reinforced cement material durability and by Koerner et al. [39] to predict the degradation of thin geosynthetic polymeric materials used in landfill liners and covers. When used to model degradation of FRP composites, two assumptions are made [40]: (1) degradation is dominated by a single chemical mechanism that is independent of exposure duration, and (2) the conditioning of the FRP bar at elevated temperatures (below the matrix glass-transition temperature) does not change the degradation mechanism when subjected to service temperatures. In other words, the degradation mechanism is independent of exposure temperature below the glass transition temperature.

Let *m* be the degradation degree or percentage, then *m/k* represents the time *t* needed to reach *m*. Accordingly, one can write

(2) $$\ln(t)=\ln\left(m/k\right)=\ln\left(m/A\right)+\frac{E_{a}}{R}\frac{1}{T}$$

In Equation (2), time *t* is the dependent variable while temperature *T* is the independent variable. The logarithm of the time needed for a material to reach a certain degree of degradation, such as 50% or 20%, is a linear function of 1/*T* with the slope of the line equal to *E*_*a*_/*R*. Theoretically, the degradation degree has no influence on *E*_*a*_/*R* because the slope only depends on the activation energy, which is independent of temperature or time. In other words, based on principles of chemistry, activation energy is constant for a given FRP bar undergoing a single degradation reaction, a fact that is reflected by the Arrhenius equation. 

**Figure 1:**
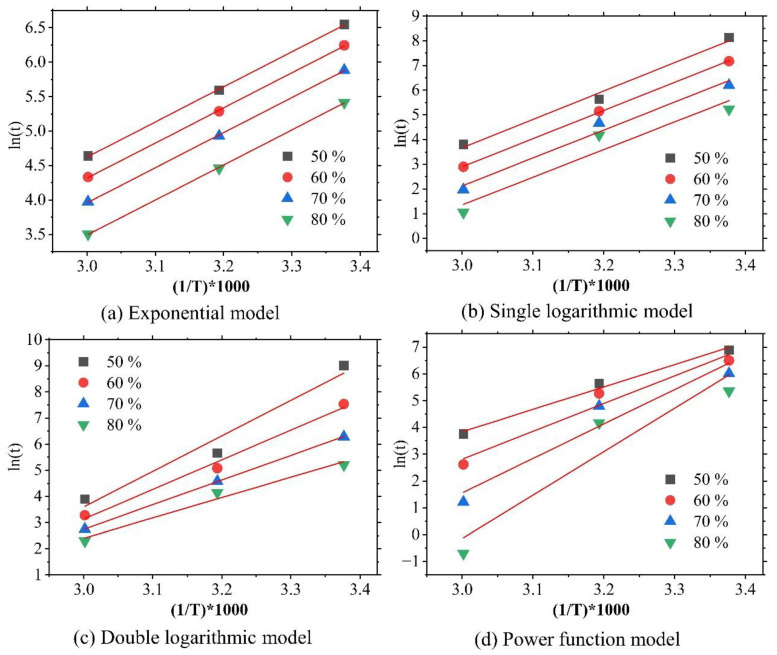
Arrhenius diagrams based on four models fitted to different percentages of retained tensile strength based on the test data of Wang et al. [41].

Equation (2) serves as the theoretical basis of the plots in the Arrhenius diagram in Figure 1. Whether the lines fitted to the data in this figure are parallel or not is commonly utilized to assess the model’s compliance with the Arrhenius equation. If they are parallel, then the degree of degradation predicted by the model is believed to comport with the Arrhenius equation. However, parallelism does not mean that the model can accurately predict the actual degree of degradation. 

Let us now assume that the time needed to reach the same degradation degree *m* under constant exposure temperatures *T*_1_ and *T*_2_ is *t*_1_ and *t*_2_, respectively. Then,

(3) $$t_{1}=\frac{m}{k_{1}}=\frac{m}{A}\exp\left(\frac{E_{a}}{R}\frac{1}{T_{1}}\right)$$

(4) $$t_{2}=\frac{m}{k_{2}}=\frac{m}{A}\exp\left(\frac{E_{a}}{R}\frac{1}{T_{2}}\right)$$

If the pre-exponential *A* in the Arrhenius equation is assumed to be temperature-independent, then division of Equation (3) by (4) gives

(5) $$\mathrm{TSF}=\frac{t_{1}}{t_{2}}=\exp\left(\frac{E_{a}}{R}\left(\frac{1}{T_{1}}-\frac{1}{T_{2}}\right)\right)$$

In current practice, typically, after obtaining *E_a_/R* based on the Arrhenius plot, the time shift factor (TSF) [7] value obtained from the accelerated tests is employed to estimate the time corresponding to a given degradation degree under long-term service temperatures.

#### 1.2.2. Time Dependency

Time dependence of FRP bar degradation is reflected by the models. The EP, SL, DL and PF are the four models widely used to predict the loss in strength of FRP bars under prescribed exposure conditions [6,8,9,10].

(1) The Single Logarithmic (SL) Model

Litherland et al. [38] first proposed the SL model, expressed as Equation (6), and used it to investigate the durability of glass-fiber reinforced cement materials. Subsequently, Bank et al. [40] applied it to FRP composites. They specified the highest and lowest exposure temperature as 0.8 times the glass transition temperature and 40 °C, respectively. This model has been also applied by several researchers [7,29,33].

(6) y=alog(t)+b

where *y* is the strength retention (%), *t* is the exposure time, *a* and *b* are model parameters. Based on this model, no parallelism in the Arrhenius diagram was reported by some researchers [8,9,10,17]. In addition, the model has some intrinsic limitations [10]. First, it is purely phenomenological. It is obtained by fitting test data to a mathematical function without being underpinned by any hypothesis regarding the associated degradation mechanism (s). Secondly, the strength retention based on Equation (6) approaches infinity at time zero.

(2) The Double Logarithmic (DL) Model

The DL model is expressed as Equation (7). The *Fib bulletin 40-2007* [42] used the constant slope of the strength retention vs. time based on Equation (7) as the standard reduction of tensile strength per decade due to environmental influence. More refinements to capture the effects of moisture, temperature, service life, diameter of FRP bar, and material type on the tensile strength retention were proposed in the *Fib bulletin 40-2007* [42].

(7) $$\log\left(y\right)=a\log(t)+b$$

This equation has been applied by several researchers [27,32,43], and in some cases, (Ruiz [8] and Weber [32]), the lines in the Arrhenius plot for different degradation degrees were found not to be parallel. The lack of parallelism would violate the Arrhenius equation, which is considered inviolable.

(3) The Power Function (PF) Model

The PF model, expressed by Equation (8), was proposed by Davalos et al. [10].

(8) $$y=100{\left(1-jt^{a+1}\right)}^{2}$$

where *j* and *a* are model parameters. It is assumed that the ultimate tensile load capacity of GFRP bars is proportional to the area of the part of the cross-section that is not permeated by the immersing or conditioning solution. When *a* = 0.5, Equation (8) becomes equivalent to the model represented by Equation (9), which is based on moisture absorption [18]. Note that Equation (8) is easier to apply than Equation (9).

(9) $$y=100{\left(1-\frac{\sqrt{2DCt}}{r_{0}}\right)}^{2}$$

In the above, *D* is the diffusion coefficient, *C* is the concentration of the conditioning solution, and *r*_0_ is the radius of the FRP bar. Equation (8) is phenomenological and must be calibrated for each bar type and size, while Equation (9) is based on moisture-absorption theories, and it identifies the parameters of the model.

(4) The Exponential (EP) Model

In 1986, based on ultrasonic and flexural test results on laminates immersed in water at 100, 60 and 40 °C, Phani and Bose [44] proposed the EP model, expressed as Equation (10), to capture the relationship between the FRP post-exposure strength and exposure time.

(10) $$y=\left(100-Y_{\infty }\right)\exp\left(-t/\tau \right)+Y_{\infty }$$

where *Y*_∞_ is strength retention at time ∞, and *τ* is the model parameter. 

In 1987, Phani and Bose [45] obtained the flexural strengths of laminates after exposure to distilled water at 42, 50, 60, 80, 90 and 100 °C, and Equation (10) was used to estimate strength at any other temperature within the above temperature range. It should be noted that, based on work by Phani and Bose [44,45], *Y*_∞_ for FRP laminates exposed to different temperatures remains practically constant, while for FRP bars this quantity varies greatly with exposure temperature. Consequently, the application of Equation (10) to FRP bars may not be appropriate. As stated by Lu et al. [36]_,_ the model precision will be influenced by the value of *Y*_∞_. In addition, it is impossible to know the tensile strength after an infinite exposure time based on limited exposure-time test data [34]. As reported by Morales et al. [28], the value of *Y*_∞_ is dependent on exposure temperature, and varies from 70.0% to 98.7%. Based on the experimental data reported by Davalos et al. [10], values of the parameters of Equation (10) are calculated by the writers as shown in Table 1. With reference to the table, *Y*_∞_ varies from 38% to 75%, showing huge variation. If TSF is used to extrapolate result of the experimental data obtained from high-temperature tests to low-temperature conditions, the extrapolated strength retention may be smaller than the predicted *Y*_∞_ obtained, based on low-temperature test results. This will lead to conflict between the extrapolated data and the *Y*_∞_ values.

In 2006, Chen et al. [46] modified Equation (10) as shown in Equation (11). They assumed that *Y*_∞_ equals zero at time infinite, and then used the modified equation for the FRP bar tensile-strength retention prediction.

(11) $$y=100\exp\left(-t/\tau \right)$$

where *t* is the exposure time and τ is the model parameter. Equation (11) has been also used by others to predict FRP bars’ retained tensile [9,34,35,36] and shear strength [30,31]. Note, in this paper, Equation (11) is termed the exponential (EP) model. The assumption of the power of *t* in Equation (11) being constant and equal to one has no theoretical foundation. We shall revisit this issue later in this paper.

#### 1.2.3. Criterion for Acceptability of Current Models

Details of the current procedure [35] for predicting the long-term strength retention of FRP bars is described below. The value of the coefficient of determination (*R*^2^) and the parallelism of the fitted lines (via regression analysis) to the transformed experimental data in the Arrhenius diagram are the principal criteria used to judge the acceptability and accuracy of a model. A value of *R*^2^ equal to 0.80 or higher is generally deemed necessary for a model to be acceptable [28,29].

**Figure 2:**
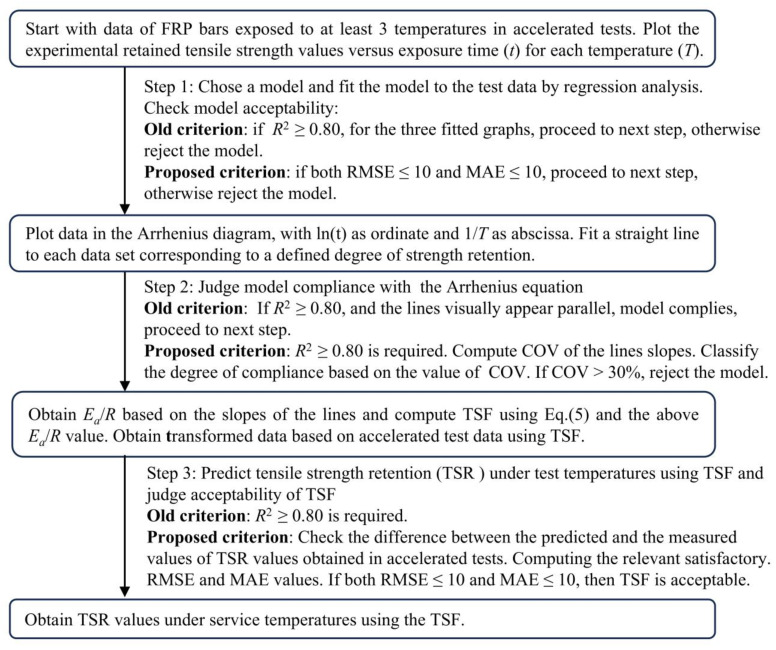
Calculation flow chart for the old and proposed criterion.

As indicated in Figure 2, the procedure involves the following steps: (1) selection of the model and fitting of the model through regression analysis to experimental data from accelerated tests conducted under different exposure temperatures. This process lets one determine the values of the model parameters. To check the goodness-of-fit, the *R*^2^ value needs to be 0.80 or higher, and if this is the case, the model is deemed acceptable. If the opposite happens, the model is rejected and a new model is sought. (2) After acceptance of the model based on Step (1), the exposure time required for strength retention values of 50%, 60%, 70% and 80% (see Bank et al. [40]) under different exposure temperatures is calculated using the model obtained in Step (1). The calculated times are plotted in an Arrhenius diagram with 1/T as the abscissa and ln(t) as the ordinate. If the lines corresponding to the preceding retained-strength percentages appear straight and parallel to each other, they are considered indicative of the correctness of the assumption that essentially one chemical reaction or corrosion mechanism is controlling the strength degradation [38]. Next, the *E_a_*/*R* value in Equation (2) is assumed equal to the slope of the parallel lines, and the *R*^2^ for the *E_a_*/*R* values based on each fitted line is calculated. If the *R*^2^ values are all 0.80 or higher, and the straight lines are deemed visually parallel, the model is accepted. If it is less than 0.8, or the straight lines are deemed not parallel, the model is rejected and a new model is adopted. (3) The TSF between the accelerated test temperatures and the service temperatures is calculated using Equation (5), where the *E_a_*/*R* value obtained in Step (2) is used. The strength-retention data from different accelerated test temperatures are thus transformed to strength-retention values under service temperatures. The transformed values are used to conduct linear regression analysis based on the chosen model. As in Step (1), *R*^2^ is again used to evaluate the goodness-of-fit. The acceptable value of *R*^2^ needs to be 0.80 or larger. Once the latter condition is satisfied, the model can be used to obtain the relationship between strength retention and exposure time under service temperatures.

## 2. Materials and Methods

### 2.1. The Proposal of Modified Exponential Model

Degradation of fiber, resin, and fiber–resin interface is the primary cause of FRP deterioration [6]. Resin degradation is mainly caused by plasticization and hydrolysis reactions. The OH^−^ etching leads to the degradation of glass or basalt fibers in an alkaline environment. The resin degradation or fiber corrosion [14] leads to damage to the fiber–resin interface [6]. 

Given the great similarities in their composition and structure, basalt and glass fibers may have similar degradation mechanisms [6,35]. The corrosion of glass typically results in the formation of a surface alteration layer [47]. The formation of this layer involves several coupled mechanisms, including hydration, hydrolysis, dissolution, diffusion, ion exchange, adsorption, and/or crystal nucleation and growth [47]. The direct precipitation of amorphous silica from the immersing solutions is assumed to be the reason for the formation of the surface alteration layer [47]. In addition, nucleation and crystallization rate, which is captured by the Avrami–Erofe’ev [48] model, could serve as one of the rate-controlling mechanisms of glass corrosion [15]. Water sorption in epoxy is believed to follow Fickian or non-Fickian diffusion [49]. Strength retention of the FRP bar has been also related to the bar mass change [16] and diffusion coefficient [17]. 

The Avrami equation, shown in Equation (12), originated in classical nucleation theory,

(12) $$f=1-\exp(-kt^{n})=1-\exp\left[-{\left(t/\tau \right)}^{n}\right]$$

where *f* is the volume fraction of the new phase, *k* is the rate constant, $\tau =k^{-1/n}$ is termed the time constant and *n* is the Avrami exponent. Equation (12) is widely used for predicting phase transformations in the field of materials science [50]. It is also used to infer the mechanism of phase transformations in polymers and inorganic glasses [51,52] and for assessing the kinetics of diffusion-controlled precipitation reactions [53].

In view of the foregoing FRP degradation mechanisms and the application areas of the Avrami equation, the writers hold the opinion that it could also be used as the theoretical foundation for a model to predict the FRP long-term durability. From the mathematical perspective, the main difference between Equation (12) and Equation (11) is the value of power *n*, which deemed variable in Equation (12) and equal to one in Equation (11). The writers do not see any basis for the assumption that the power of *t* in Equation (11) is always one. To validate this belief, linear regression analysis was conducted on a set of experimental data [33] by fitting Equation (11) and Equation (13) to them. Note, in this work Equation (13) is proposed and is termed the modified exponential (MEP) equation. The computed values of the two models’ parameters, as well as their relevant *R*^2^ values, are shown in Table 2.

(13) $$y=100\exp(-t^{n}/\tau )$$

As shown in the penultimate column of Table 2, *n* can significantly deviate from one. This is the justification for the introduction of the MEP model in this work.

### 2.2. The Proposal of Recommended Procedure for Evaluating Goodness-of-Fit for Traditional Models

#### 2.2.1. Analysis of the Parallelism of Fitted Lines in the Arrhenius Diagram

As discussed earlier, a key criterion for the acceptability of the traditional models of FRP durability is the parallelism of the ln(*t*) versus 1/*T* lines for defined degrees of strength retention fitted to the corresponding data. The slope of the lines is *E_a_*/*R*, which is theoretically constant because the activation energy for a given type of FRP is independent of the retained strength, exposure temperature and duration. So, the purpose of plotting these lines is to determine (a) whether the fitted lines satisfy the condition of constant slope and stay parallel, and (b) if they are not parallel, how much they deviate from parallelism. In the following analysis, the necessary condition for the existence of parallelism is analytically/mathematically specified. 

Let *y* represent the retained strength, and *t_i_* the exposure time needed to reach *y* under exposure temperature *T_i_* (*i =* 1,2). Under these conditions, the slope of the lines in the Arrhenius diagram based on the SL DL and PF models, (see Equations (6), (7) and (8), respectively), can be written in the form of Equation (14), Equation (15), and Equation (16), respectively. As the latter set of equations show, when *a*_1_ = *a*_2_, the slope has no relationship to the retained strength, *y*, and thus, theoretically, only under this condition would the lines be parallel. The condition *a*_1_ = *a*_2_ is practically impossible to attain using physical test data. Consequently, in practice, parallelism cannot be assured based on these models.

(14) $$\mathrm{slope}=\frac{\ln\left(t_{1}\right)-\ln\left(t_{2}\right)}{1/T_{1}-1/T_{2}}=\frac{1}{1/T_{1}-1/T_{2}}\left(\frac{y}{a_{1}}-\frac{y}{a_{2}}+\frac{b_{2}}{a_{2}}-\frac{b_{1}}{a_{1}}\right)\ln10$$

(15) $$\mathrm{slope}=\frac{\ln\left(t_{1}\right)-\ln\left(t_{2}\right)}{1/T_{1}-1/T_{2}}=\frac{1}{1/T_{1}-1/T_{2}}\left(\frac{\mathrm{lg}y}{a_{1}}-\frac{\mathrm{lg}y}{a_{2}}+\frac{b_{2}}{a_{2}}-\frac{b_{1}}{a_{1}}\right)\ln10$$

(16) $$\begin{array}{l} \mathrm{slope}=\frac{\ln\left(t_{1}\right)-\ln\left(t_{2}\right)}{1/T_{1}-1/T_{2}}\\ =\frac{1}{1/T_{1}-1/T_{2}}\left[\left(\frac{1}{a_{1}+1}-\frac{1}{a_{2}+1}\right)\ln\left(1-\sqrt{y/100}\right)+\frac{\ln j_{2}}{a_{2}+1}-\frac{\ln j_{1}}{a_{1}+1}\right] \end{array}$$

As for the EP model, represented by Equation (11), the slope can be expressed as Equation (17), which shows no relationship between the slope and the retained strength *y*.

(17) $$\mathrm{slope}=\frac{\ln\left(t_{1}\right)-\ln\left(t_{2}\right)}{1/T_{1}-1/T_{2}}=\frac{\ln\left(\tau_{1}/\tau_{2}\right)}{1/T_{1}-1/T_{2}}$$

Accordingly, the ln(*t*) versus 1/*T* lines corresponding to different *y* values plotted in the Arrhenius diagram would be automatically parallel. This means that it is not necessary to check the parallelism of the lines based on the EP model. Alternatively, for this model, checking the parallelism requirement is superfluous [8].

As for the MEP model, represented by Equation (13), the slope can be expressed as Equation (18). In this case, when *n*_1_ = *n*_2_, the slope is independent of retained strength. Hence, as in the case of the SL, DL and PF models, when *n*_1_ = *n*_2_, there is no need to check the parallelism of the fitted lines in the Arrhenius diagram for different *y* values.

(18) $$\mathrm{slope}=\frac{\ln\left(t_{1}\right)-\ln\left(t_{2}\right)}{1/T_{1}-1/T_{2}}=\frac{\frac{1}{n_{1}}\ln\tau_{1}-\frac{1}{n_{2}}\ln\tau_{2}+\left(\frac{1}{n_{1}}-\frac{1}{n_{2}}\right)\ln\left(\ln\frac{100}{y}\right)}{1/T_{1}-1/T_{2}}$$

#### 2.2.2. Discussion of the Parameter Used to Judge Parallelism

As discussed in Section 3.2, for the EP model, mathematically, parallelism automatically exists. For the other four models, parallelism is virtually impossible. The current practice is to fit the lines to the experimental data in the Arrhenius diagram and visually assess whether the lines appear parallel [7,8,9,29,30,31,32,33,34,35,36]. As assessing parallelism visually is subjective, the writers propose the coefficient of variation (COV) of the slope of the lines as a consistent and objective criterion for assessing the level of parallelism. Theoretically, perfect parallelism exists only when the COV of the slopes is equal to zero, which is practically impossible. Consequently, it is necessary to define an upper limit for the COV that could be deemed acceptable for assuming parallelism. Based on the analyses performed in the current study, tentatively, a COV of 30% is set as the upper limit for acceptability. Accordingly, the degree of parallelism can be considered good for a COV of 10% or lower, moderate for 10% to 20%, and low or poor for larger than 20% but less than 30%. Anything above 30% would indicate the absence of an acceptable degree of parallelism and the rejection of the associated model. It should be noted that the COV has no influence on the model parameters and predicted results; it is only used to check whether the fitted lines have a constant E_a_/R value as required by the Arrhenius equation.

#### 2.2.3. Proposed Parameters for Evaluating Goodness-of-Fit

Traditionally, *R*^2^ is the only parameter used to measure the goodness-of-fit, but *R*^2^ is not suitable for nonlinear models [54]. Although the models discussed earlier can be linearized, nevertheless, the use of *R*^2^ alone to assess their acceptability could be problematic. For example, one can fit the model predicted retained-strength values versus exposure period under a given exposure temperature to the corresponding experimental data and compute the companion *R*^2^ value. Since in current practice, at least three exposure temperatures must be used to validate the model, this will result, in practically all cases, in three different values of *R*^2^. Suppose two of the values satisfy the acceptability criterion but the third one does not: would the model be accepted or rejected? There is no obvious answer to this question. Hence, the use of *R*^2^ alone is not sufficient to judge the acceptability of a model. As described below, the writers propose that the *R*^2^ criterion be supplemented by additional metrics of acceptability.

The writers recommend that the Root Mean Square Error (RMSE) and the Mean Absolute Error (MAE), given by Equations (19) and (20), respectively, be used as the metrics for judging the acceptability of the goodness-of-fit of the FRP bar durability model.

(19) $$RMSE=\sqrt{\frac{{\sum_{i=1}^{n}\left(S_{pi}-S_{ti}\right)}^{2}}{n}}$$

(20) $$MAE=\frac{\sum_{i=1}^{n}\left|S_{pi}-S_{ti}\right|}{n}$$

where *S_pi_* and *S_ti_* are the *ith* predicted and experimental values of the retained strength, respectively, *n* is the total number of data points used in the fitting, and *i* = *1*, *n.* It is recommended that for acceptability of the fit, both metrics should be smaller than 10. These recommendations are based on the current practices in similar situations in other areas of engineering studies, as described below. 

To estimate the shear capacity of concrete elements reinforced with FRP [21,55], the statistical parameters Root Mean Square Error (RMSE), Mean Absolute Error (MAE), and Mean Absolute Percentage Error (MAPE) are used as metrics to evaluate the precision of machine learning models. To predict the tensile strength retention of FRP bars, RMSE and MAE are used to evaluate the accuracy of artificial intelligence (AI)-generated models, such as the optimized tree-based random forest model [21], computational AI prediction models [24], and non-linear genetic-based models [23]. Since MAPE is an alternative form of MAE, it suffices to use either one of them. In these AI models [21,23,24] for FRP bar tensile-strength retention, the maximum value of MAE and RMSE are 8.4 and 9.2, respectively. Here, for simplicity, both metrics are required to be less than 10. It is important to point out that for a given set of data, RMSE and MAE will have a single value, contrary to the multiple values of *R*^2^ for the same set of data as mentioned earlier.

#### 2.2.4. Recommended Procedure for Evaluating Goodness-of-Fit for Traditional Models

With reference to Figure 2, first, the model type to be used must be selected and regression analysis must be performed using the experimental data corresponding to different test temperatures. Then, the RMSE and MAE values involving all the predicted and the companion test values must be calculated. It should be emphasized that, regardless of the number of test temperatures used in the experiment, RMSE and MAE will each have a single value. If their values are both smaller than 10, then one can proceed to the second step; otherwise, the model must be rejected and a new model must be selected. 

In the second step, the required exposure time for strength retention to reach 50%, 60%, 70% and 80% (referred to by Bank et al. [40]) under different test temperatures needs to be calculated using the selected model. The calculated values must be plotted in the Arrhenius diagram and the associated *R*^2^ should be used to evaluate the goodness-of-fit. If all the *R*^2^ are equal or greater than 0.80, then the fit is deemed acceptable. The COV should be used to judge the difference among the slopes of the lines corresponding to different degrees of retention. A COV value of up to 30% can be deemed acceptable for assuming the parallelism of the lines. If both the *R*^2^ and COV values satisfy the foregoing limits, then the computed *E_a_/R* can be assumed to be satisfactory. If the value of either parameter exceeds the specified limit, then the chosen model is not satisfactory, and one must choose a new model and repeat the process until the above criteria are met.

Once Step 2 is successfully completed, in the third step, the TSF that maps the retained strength values under the accelerated test temperatures onto the target service temperatures can be calculated by inserting the computed *E_a_*/*R* value in Equation (5). In this manner, all the test data for different accelerated test temperatures could be transformed to the service temperature condition. Thereafter, the transformed data can be fitted to obtain the new model parameters under service temperatures. Then RMSE and MAE can be calculated using the test data and their predicted values using the new model parameters. If RMSE and MAE are not both smaller than 10, then the model is not acceptable. 

### 2.3. Experimental Database

#### 2.3.1. Establishment of Experimental Database

Tensile strength-retention values are used to evaluate the degradation degree of FRP bars, as tensile strength is the sole mechanical parameter utilized in ASTM D7705/D7705M-12 [56], and is asserted by Wang et al. [57] to be a reliable measure of FRP bar durability. In practice, not all published papers provide numerical values of the data needed for analysis. Instead, some data are presented in graphical form. In such cases, in the current study, WebPlotDigitizer was utilized to extract the numerical values of the data points in the graphs. To ensure data accuracy, if the points in the graphs were difficult to distinguish, the data were excluded from the database. 

The software 1stOPT 10.0 [58], developed by 7D-Soft High Technology Inc., was utilized to obtain the value of the model parameters. For the FRP bar, the initial strength retention is generally assumed to be 100%, and it could be used as the supplied data for model regression. While for the SL and DL models, the domain of definition must be greater than zero, for the EP and PF models, strength retention theoretically equals 100% at exposure time zero. So, (0, 100) could be used as a supplied data point for the latter models. For regression analysis involving the two logarithmic models, at least three data points (i.e., three strength retention values) are needed for each exposure temperature, while for the EP and PF models, a minimum of two data points is necessary. For ease of comparison, in the present research, a minimum of three data points is used in all cases. In addition, in the Arrhenius plot, at least three temperatures are needed [9]. Hence, in the current database, the number of exposure temperatures in accelerated tests and the number of retained strength values for each temperature are not fewer than three. 

For uniformity, all exposure times are converted to days. If the exposure time is reported in months, as in papers [41,59,60,61,62], a month is assumed to have 30 days. If the exposure temperature is described as room temperature, as in paper [61], and the actual value of the temperature is not given, it is assumed to be 25 °C.

In addition, the following notations are applied in the current analysis. Letters P, V, and E designate polyester, vinyl ester and epoxy matrix, respectively. Notations E-g, ECR-g, G, B and C designate E-glass, ECR-glass, glass, basalt and carbon fiber, respectively. G is used to describe glass in the generic sense when the original researchers have not specified the specific type of glass. As for the fiber fraction, generally either fraction by mass or by volume is specified; hence, here ‘m’ signifies fiber fraction by mass, and ‘v’ by volume. For example, 73.3 m denotes a fiber fraction which is 73.3% by mass. In summary, a typical designation would read E-g/V/12.7/73.3 m, which describes an FRP bar made of E-glass fibers with a vinyl ester matrix, having 12.7 mm diameter and a fiber fraction of 73.3%, by mass. 

As for the immersing solution, AS, ASS and S represent alkaline, alkaline salt, and salt solution, respectively, while TW, SW and DI designate tap water, sea water, and deionized water, respectively. Some researchers have performed durability tests by embedding the bars in mortar or concrete, in which case ‘C’ and ‘M’ refer to concrete and mortar, respectively. Most conditioned bars have been tested in unstressed condition, but in some cases, bars were stressed, so S## is used to represent the bar stress level. For example, S0.40 represents a bar stressed to 40% of its guaranteed or ultimate tensile strength. AS/12.8/S0.40 denotes a bar stressed to 40% of its tensile strength and immersed in an alkaline solution, with pH of 12.8. If the bar is embedded in seawater concrete, it is designated by SC, while SW represents a bar embedded in normal concrete and immersed in seawater. 

As for the exposure temperature, time, and the corresponding tensile strength-retention value, as shown in Appendix A, the relevant data are shown in the column with a heading of *T*/*t*/TSR. In this column, the specification 60/188/67.4 refers to a bar exposed to 60 °C for 188 days, having tensile strength retention of 67.4%.

#### 2.3.2. Database Features

**Figure 3:**
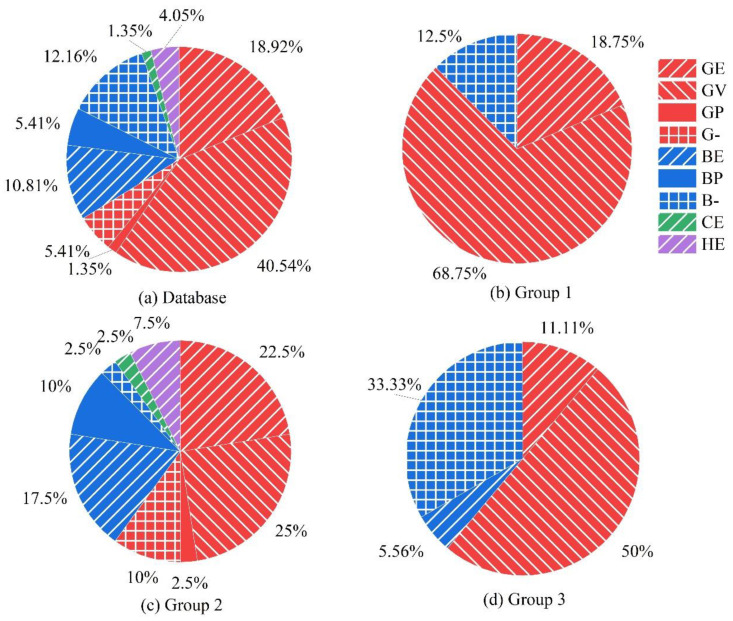
Proportion of different types of FRP bars in the database.

As mentioned earlier, the EP model is currently the most popular, but, as discussed, it has some deficiencies. To rectify those deficiencies, the MEP model is proposed in the current study. To verify the proposed MEP model, the writers have compiled tensile strength-retention results of FRP bars from 29 related papers published from 2008 to 2024. The information regarding FRP bars, exposure conditions, and corresponding tensile strength-retention values for different exposure conditions is shown in Appendix A. The collected database comprises 74 groups (903 data points); each group represents one type of bar exposed to a specific environment under different temperatures and for different exposure periods. These stressed or unstressed bars were exposed to various environments, including none-alkaline solution (shown in Table A1, named sub-database 1), alkaline solutions (shown in Table A2, named sub-database 2), and embedded in mortar or concrete (shown in Table A3, named sub-database 1). The proportions of each type of FRP bar in each group are shown in Figure 3. As shown in Figure 3a, the FRP bars in the above works primarily consist of GFRP (66.22%) and BFRP (28.38%) bars, with some CFRP (1.35%) and hybrid (4.05%) bars. 

As for the bars exposed to non-alkaline solutions (i.e., sub-database 1), there are 16 groups (187 data points) in total; 14 groups (169 data points) are unstressed and 2 groups (18 data points) are stressed. As shown in Figure 3b, they comprise GFRP (87.5%) and some BFRP (12.5%) bars. For the test specimen’s classification based on the immersing alkaline solution (i.e., sub-database 2), there are 40 groups (479 data points) in total. As shown in Figure 3c, they mainly consist of GFRP bars (60%), some BFRP (30%), CFRP (2.5%) and hybrid bars (7.5%). Considering the FRP bars embedded in mortar/concrete (i.e., sub-database 3), they are divided into 18 groups (237 data points), where 16 groups (217 data points) are unstressed and 2 groups (18 data points) are stressed. As shown in Figure 3d, the 18 groups mainly consist of GFRP (61.11%) and BFRP (38.89%) bars.

## 3. Results and Discussion

Computed values of the parameters of the different models based on the accelerated test data are shown in Appendix B. The discussion in this section is based on the results presented in this appendix. 

### 3.1. Comparison Between the Existing Models and the Proposed Model Acceptability Criteria

**Figure 4:**
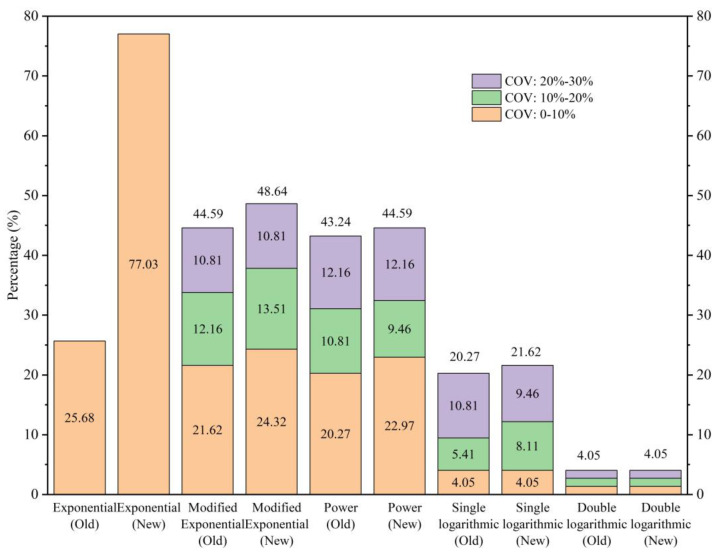
Percentage of groups that reached the required criterion using the old and new criterion based on five models.

In the present research, there are 74 groups of experimental data, and each group of data is analyzed using the five FRP durability models discussed earlier in this paper. The analysis entails the application of the prevailing procedure versus the proposed procedure for the acceptability of a model. It should be noted that both procedures use the COV as the criterion for judging parallelism of the lines in the Arrhenius diagram. The percentage of groups satisfying this criterion is shown in Figure 4. The number of groups meeting the old or new criterion based on different models is shown in Table 3. Table A9 lists every group and whether it meets or does not meet the old or new criterion, based on different models.

As shown in Figure 4, when the existing procedure is used, the percentages of groups satisfying the criterion based on each model are, in descending order, MEP (44.59%), PF (43.24%), EP (25.68%), SL (20.27%), and DL (4.05%). When the proposed procedure by the writers is used, the corresponding percentages, in descending order, are the EP (77.03%), MEP (48.64%), PF (44.59%), SL (21.62%), and DL (4.05%). The DL model exhibits the lowest percentage of acceptability using either procedure. The EP model exhibits the highest percentage when the proposed procedure is used, while the MEP model exhibits the highest percentage when the existing procedure is used. 

Comparing the results of the two procedures applied to the same model, for the EP model, the proposed procedure increased the percentage of cases satisfying the criterion from 25.68% to 77.03%. Insofar as the EP model is concerned, the COV of all 74 groups equals zero. In addition, as shown in Table 3, for this model, the proposed procedure not only agrees with most of the results of the existing procedure, but it further adds to the percentages of cases meeting the acceptability criterion. 

In the case of the DL model, the two procedures show the same number of cases meeting the acceptability criterion. As for the MEP, the PF, and the SL models, compared to the existing procedure, the proposed procedure finds a slightly higher percentage of cases meeting the acceptability criterion. In addition, as shown in Table 3, for the MEP, SL, DL and PF models, the proposed and the existing method show similar percentages of acceptability.

### 3.2. Influence of Data Aberration on the Different Models’ Results

Theoretically, strength retention of an FRP bar exposed to the same environment is expected to decrease with the increase in exposure time. However, based on the experimental results in the present database, the retained strength values for some groups do not monotonically decrease. If the predicted retained strength increases with the increase in exposure time, it should be considered an aberration. If the data pertaining to these aberrations are used for fitting the model, the model could also show increase in retained strength with increase in exposure time. The exclusion of the aberrational experimental data from the regression analysis will reduce the number of data points for meaningful fitting and may result in a COV that would not be able to meet the requirement of parallelism in the Arrhenius diagram.

Considering the occurrence of the preceding phenomenon in the cases included in the current database, the number of groups that shows this aberration in sub-database 1, 2, and 3 is 13, 14 and 8, respectively. As shown in Table 4, the experimental data in 81.25% of groups in sub-database 1 exhibit the aberration. Compared to sub-database 2 and 3, sub-database 1 more clearly exhibits the above phenomenon. Due to the lower pH of the non-alkaline solution in which the specimens in sub-database 1 were immersed compared to the pH of the alkaline solutions in which specimens in sub-database 2 and 3 (embedded in mortar or concrete) were immersed, the phenomenon could be ascribed to this difference between the pH values of the immersing solutions.

The groups in which the predicted strength retention increase with exposure time are shown in Table 5. Notice that, when the models are applied to all the 35 groups exhibiting the aberration, the EP model predicts only one group (2.86% of the 35 groups), the MEP model seven groups (20%), the SL and DL models eight groups each (22.86% each) and the PF model nine groups (25.71%) having increased strength retention with exposure time. This phenomenon seems to have little effect on the results of the EP model compared to those of the other four models. For Group 1–5, when the EP model is used, the value of the parameter τ (see Equation (5)) is negative. These are the only groups for which τ is negative, but, theoretically, the value of *τ* in this model cannot be negative. 

### 3.3. Model Comparison

#### 3.3.1. Models’ Parameters Characteristics

In Section 3.1, groups based on different models meeting the existing and proposed acceptance criteria were identified, but the predicted strength retention after exposure to service temperatures remains unknown. On the other hand, the main purpose of developing a model is to predict the exposed-bar retained strength under field conditions. Hence, to compare the predicted retained strength values given by the different models, groups meeting the existing and proposed criteria are taken into consideration. It should be noted that the existing and proposed criteria do not change model parameter or predicted strength-retention results. In the current work, groups satisfying the existing or the proposed criteria are analyzed. First, model parameters for exposure temperatures of 5 °C, 10 °C, 15 °C, 20 °C, 25 °C and 30 °C are obtained, and then the corresponding retained tensile strength values after 1, 5, 10, 25, 50 and 100 years of exposure to the same temperatures are calculated. Due to the logarithmic form of the EP models, all the predicted strength retention values given by these models range between 0 and 100%. But the predicted strength retention based on SL, DL and PF models may be significantly larger than 100 or smaller than 0, which is obviously incorrect. For simplicity of classification, herein, predicted strength retention values larger than 110% or smaller than 0% are classified as wrong. When the DL model is used, all the three groups shown in Table 3 fall into the “Right” classification. When the SL model is applied, half of the 18 groups fall within the same classification. When the PF model is applied, 18 of the total 35 groups fall within the preceding classification. In addition, the predicted strength retention values by the SL and DL models always decrease with the increase of exposure time, while those predicted by the PF model fluctuate as the exposure time increases. 

In the writers’ opinion, a model is incorrect if its predicted strength retention is larger than 110% or smaller than 0% or if the strength retention values arbitrarily fluctuate with increase in exposure time. According to this yardstick, model parameters for bars exposed to 5 °C, 10 °C, 15 °C, 20 °C, 25 °C and 30 °C are shown in Appendix D (i.e., from Table A10, Table A11, Table A12, Table A13 and Table A14). It should be noted that the model parameters for bars exposed to 5 °C, 10 °C, 15 °C, 20 °C, 25 °C and 30 °C were obtained by transforming the experimental data using TSF. As shown in Appendix D, parameter *n* of the MEP model, and *a* of the SL, DL and PF model is the same for the same bars exposed to different temperatures. This means if the predicted data are plotted in the Arrhenius diagram, all the fitted lines will be parallel as per the mathematical analysis in Section 3.2, and the COV of the slopes of the lines will be zero. Parameter *τ* of the EP and the MEP models, and parameter *b* of the SL and DL would decrease with the increase in exposure temperature, and parameter *j* of the PF model would increase with the increase in exposure temperature. The ranges of these parameters are shown in Table 6.

#### 3.3.2. Identification of the Groups of Data Satisfying All the Models Discussed

**Figure 5:**
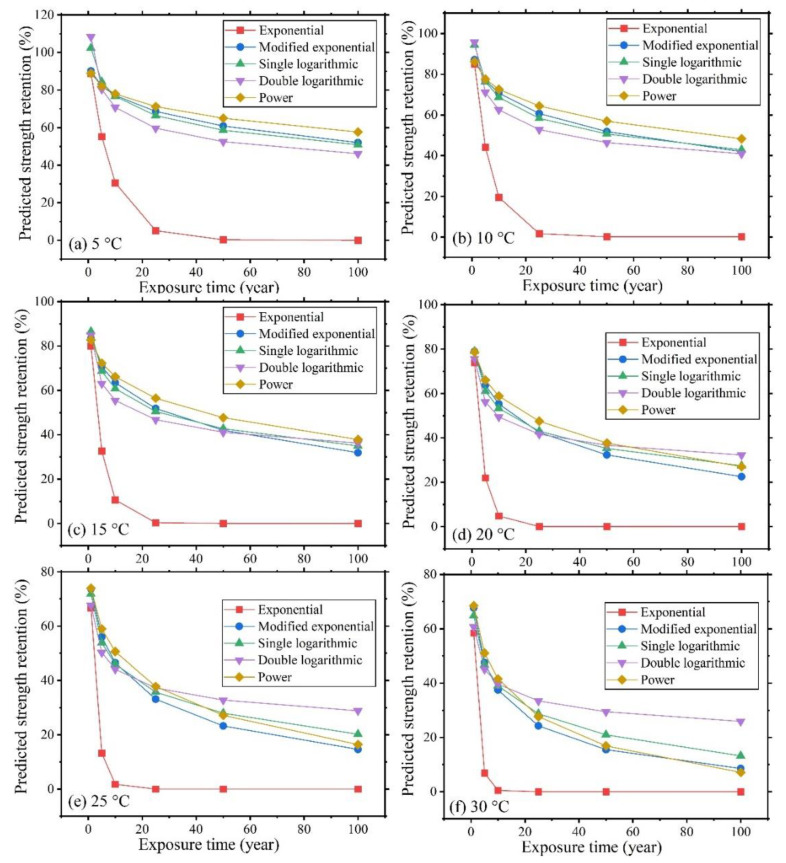
Predicted strength retention of Groups 2–28 based on the five models.

As shown in Table 3, taking the predicted strength retention range from 0 to 110% into consideration, only Groups 2–28 meet this requirement in the case of all five models discussed earlier. The predicted strength retention values of Groups 2–28 based on the five models are shown in Figure 5.

As shown in Figure 5, the predicted strength retention values by the EP model are lower than those predicted by the other models. Overall, the predicted values of the other four models are relatively close. The *E_a_*/*R* values based on the EP, MEP, SL, DL, and PF models are 5092.30, 11,178.20, 11,347.00, 10,541.23, 11,944.83, respectively. Notice that the values given by the last four models are relatively close to each other, while the one given by the EP model is much smaller. These results are based on only one group. Below, further comparisons will be made.

#### 3.3.3. Comparison of Strength Retention Values Predicted by the Different Models

There is no known or verifiable long-term strength retention value that could be used as reference to measure the accuracy of the different models. Here RMSE is used to evaluate the difference between the predicted strength retention values based on any two of the above models. The computed results are shown in Table 7. Notice that the mean value of RMSE involving the EP model and any other model is much larger than the values obtained for pairs of the other models. This shows that the predicted strength retention based on the EP model is significantly different and much smaller than those predicted by the other models. The mean value of RMSE for the other pairs of models is smaller than 15, which signifies that the latter models’ predicted retained-strength values are relatively close to each other. The same basic results are yielded when the pertinent data for Group 2–28 are used.

#### 3.3.4. Comparison of E_a_/R

Once again, the RMSE is computed to compare the difference between the *E_a_*/*R* values for different pairs of the above models. Detailed *E_a_*/*R* values of different models are shown in Appendix B. The computed values are shown in Table 8. It can be observed in this table that the *E_a_*/*R* value involving the EP model is rather different (RMSE > 9000) and much smaller than those that do not involve this mode. The RMSE value based on the combination of any two models, except the combination involving the EP model, is smaller than 5000. This signifies that the *E_a_*/*R* values computed based on the other four models are relatively close to each other. Note that the values given by the MEP and PF models are comparable.

#### 3.3.5. The Influence of Parameter n of the Modified Exponential Model

As shown in Table 6, parameter *n* varies from 0.138 to 2.354. In fact, it is smaller than 1.0 for most groups (>80%). When *n* < 1.0, the predicted strength retention based on the MEP model is generally larger than that based on the EP model, and the value of parameter *τ* of the MEP model is smaller than that of the EP model. When *n* > 1.0, the predicted strength retention based on the former may be larger or smaller than that based on the latter, but the value of *τ* for the former is larger than that for the latter. The RMSE between the EP and MEP models based on the absolute value of (1 − *n*) is shown in Table 9. It can be observed that the RMSE generally exhibits an increasing trend with the increase in the absolute value of (1 − *n*).

## 4. Conclusions

Five models were used to compare the difference among the predicted tensile strength-retention values of FRP bars. They included the exponential, single logarithmic, double logarithmic, power function and modified exponential models. A database of accelerated test data showing values of FRP bars’ tensile strength retention under various exposure conditions was established. The test bars were divided into 74 groups, comprising 903 data points, which included GFRP, BFRP and CFRP bars made with different types of matrices. Numerical analyses were performed to check the extent to which the fitted lines corresponding to defined degrees of strength retention plotted in the form of ln(*t*) versus 1/*T* in the Arrhenius diagram deviate from parallelism. To evaluate the goodness-of-fit of the lines, the RMSE and MAE metrics are used, while the extent of the difference among the slopes of the lines is assessed by computing their COV. Based on the present analyses, the following conclusions are reached:

(1) When using the exponential model, there is no need to check the parallelism of the lines plotted in the Arrhenius diagram, as this is a priori satisfied by the functional form of the model. The lines in the Arrhenius diagram plotted based on the modified exponential, single logarithmic, double logarithmic, and power function model will not be parallel in the view of mathematics. The statistical parameter COV can be used for evaluating the variability of the slope values.

(2) For the modified exponential, single logarithmic, double logarithmic or power function model, when the proposed RMSE and MAE parameters are used versus *R*^2^, the number of test groups which satisfy the acceptability criterion is almost the same. The application of RMSE and MAE versus *R*^2^, significantly increases the number of groups meeting the acceptance criterion based on the exponential model.

(3) For regression results based on transformed test data using TSF, the model parameter *n* of the modified exponential model, and *a* of the single logarithmic, double logarithmic model or power model, are the same for a group of bars exposed to different temperatures. Parameter *τ* of the exponential and the modified exponential model, and *b* of the single logarithmic and double logarithmic models, decrease with the increase in exposure temperature. Parameter *j* of the power function model increases with the increase in exposure temperature.

(4) The aberration of measured data in accelerated tests could lead to unreasonable model prediction. Such aberration is found to have a minor influence on the predicted strength retention by the exponential model, while it significantly affects the predicted strength by the modified exponential, single logarithmic, double logarithmic, and power function models.

(5) The modified exponential model generally exhibits a similar *E_a_*/*R* value and predicted strength retention compared to the single logarithmic, double logarithmic, and power function models. The exponential model generally exhibits the lowest *E_a_*/*R* value, and the smallest predicted strength retention compared to the other four models. The difference in predicted strength-retention values by the exponential and the modified exponential model shows an increase with an increase in the absolute value of the parameter (1 − *n*).

## Figures and Tables

**Table 1:** Parameter values based on Equation (10).

Reference	Temperature °C	τ	*Y*_∞_	*R* ^2^
[10]	20	198.03	75.01	0.93
	40	92.80	63.82	0.94
	50	80.45	45.75	0.97
	60	69.42	43.23	0.98
[10]	20	148.94	74.29	0.85
	40	251.24	47.10	1.00
	60	104.40	37.98	0.94

**Table 2:** Regression results for fitting Equations (13) and (11) to test data.

Ref.	*T* (°C)	Time (day)	Tensile Strength Retention (%)	Equation (11)		Equation (13)		
				τ	*R* ^2^	τ	*n*	*R* ^2^
[33]	23	60	99.1	4932.96	0.97	49,615.64	1.407	0.99
		120	98					
		210	96.8					
		365	92.1					
	40	60	98.6	3619.24	0.99	3551.60	0.997	0.99
		120	96.4					
		210	94.5					
		365	90.4					
	50	60	97	3173.73	0.96	1395.80	0.854	0.95
		120	95.6					
		210	94.4					
		365	89.1					

**Table 3:** The number of groups meeting old or new criterion based on different models.

Procedure Type	Model Type				
	Exponential	Modified Exponential	Single Logarithmic	Double Logarithmic Model	Power Function
Both existing and proposed	18	33	13	3	30
Existing only	1	0	2	0	2
New only	39	3	3	0	3

**Table 4:** Percentage of groups in which data show retained strength aberration.

Sub-Database Type	Sub-Database 1	Sub-Database 2	Sub-Database 3
Percentage (%)	81.25	35.00	44.44

**Table 5:** Detailed groups in which predicted strength retention increases with exposure time increase.

Model Type	Exponential	Modified Exponential	Single Logarithmic	Double Logarithmic	Power
Detailed group	1–5	1–5,1–10,2–8,2–9,2–13,2–16,3–2.	1–5,1–10,2–8,2–9,2–13,2–16,3–2,3–5	1–5,1–10,2–8,2–9,2–13,2–16,3–2,3–5	1–5,1–6,1–10,2–8,2–9,2–13,2–16,3–2,3–5
The number of groups	1	7	8	8	9

**Table 6:** Values of each model parameter for bars exposed to 5 °C to 30 °C.

Model Type	Parameter	Min	Max
Exponential	*τ*	205.44	140,684,239.9
Modified exponential	*n*	0.138	2.354
	*τ*	6.53	199,084,913
Single logarithmic	*a*	−29.01	−4.96
	*b*	99.31	168.32
Double logarithmic	*a*	−0.513	−0.162
	*b*	2.202	2.906
Power Function	*a*	−0.862	0.238
	*j*	1.156 × 10^−8^	4.127 × 10^−2^

**Table 7:** Prediction comparison results between any two models.

Two Model Types for Comparison	Sample Size	RMSE				
		Mean	COV (%)	Std	Min	Max
Modified Exponential model and Exponential model	33	31.76	56.98	18.09	4.12	78.75
Modified Exponential model and Single logarithmic model	5	13.31	90.85	12.09	3.35	26.62
Modified Exponential model and Double logarithmic model	3	7.31	10.84	0.79	6.40	7.89
Modified Exponential model and Power function model	18	2.32	80.41	1.87	0.08	6.05
Exponential model and Single logarithmic model	7	41.81	52.22	21.84	4.42	67.12
Exponential model and Double logarithmic model	2	22.55	85.44	19.27	8.93	36.18
Exponential model and Power function model	17	43.67	31.92	13.94	20.37	78.54
Single logarithmic model and Double logarithmic model	2	2.00	5.39	0.11	4.77	4.92
Single logarithmic model and Power function model	2	5.78	21.68	1.25	4.89	6.66
Double logarithmic model and Power function model	1	9.08	-	-	9.08	9.08

Note: ‘-’ denotes the fact that no available numerical value exists.

**Table 8:** *E_a_*/*R* comparison results between any two models.

Model Pairs Used in the Comparison	Sample Size	RMSE
Modified Exponential model and Exponential model	33	9031.40
Modified Exponential model and Single logarithmic model	5	4685.24
Modified Exponential model and Double logarithmic model	3	1161.66
Modified Exponential model and Power function model	18	539.18
Exponential model and Single logarithmic model	7	18,164.80
Exponential model and Double logarithmic model	2	3854.87
Exponential model and Power function model	17	11,439.71
Single logarithmic model and Double logarithmic model	2	1108.96
Single logarithmic model and Power function model	2	4951.03
Double logarithmic model and Power function model	1	1403.60

**Table 9:** RMSE between exponential and modified exponential model classified by Abs (1 − n).

Absolute Value of (1 − *n*)	Sample Size	Mean	COV (%)	Std.	Min	Max
<0.2	6	13.02	52.42	6.83	4.12	21.47
0.2–0.4	8	21.15	63.69	13.47	7.49	46.22
>0.4	19	42.14	35.02	14.76	17.73	78.75

## Data Availability

Data are contained within the article.

## Nomenclature

*k*	the rate constant
*A*	the frequency factor or the pre-exponential in the Arrhenius equation
*E_a_*	the activation energy (kJ·mo1^−1^)
*R*	the gas constant (8.314462 J/K·mol)
*T*, *T*_1_, *T*_2_	the temperature
*m*	the degradation degree or percentage
*t*, *t*_1_, *t*_2_	time
y	the strength retention (%)
*a*, *b*, *j*, *τ*	model parameter
*D*	the diffusion coefficient
*C*	the concentration of the conditioning solution
*r*_0_	the radius of the FRP bar
*Y*_∞_	strength retention at infinite time
*f*	the volume fraction of the new phase
*n*	the Avrami exponent
**Abbreviations**	
MEP	modified exponential
SL	single logarithmic
DL	double logarithmic
PF	power function
EP	exponential
TSF	time shift factor
FRP	fiber-reinforced polymers
TSR	tensile strength retention

## Appendix A. Database

**Table A1:** Sub-database 1: bar exposure to non-alkaline solution.

Year	Group Number	FRP Type	Exposure Condition	*T*/*t*/TSR
2008 [63]	1-1	E-g/V/ 12.7/73.3 m	TW	25/30/91.6
				25/60/85.8
				25/90/80
				25/132/88.9
				40/30/89.3
				40/60/85.1
				40/90/82.8
				40/132/84.1
				80/30/85.1
				80/60/76
				80/90/79.5
				80/132/78.1
2008 [63]	1-2	E-g/V/ 12.7/69.1 m	TW	25/30/94.8
				25/60/75.7
				25/90/81.7
				25/132/87.8
				40/30/92.3
				40/60/87.1
				40/90/78.2
				40/132/81.2
				80/30/66.4
				80/60/55.3
				80/90/49.1
				80/132/43.9
2008 [63]	1-3	E-g/V/ 12.7/73.3 m	S/3%NaCl	25/30/93
				25/60/81.6
				25/90/82.5
				25/132/86.4
				40/30/90.6
				40/60/84.3
				40/90/84.4
				40/132/87.4
				80/30/89.7
				80/60/80.6
				80/90/80
				80/132/80.7
2008 [63]	1-4	E-g/V/ 12.7/69.1 m	S/3%NaCl	25/30/94.5
				25/60/84.1
				25/90/83.5
				25/132/80.8
				40/30/89.6
				40/60/77.2
				40/90/81.5
				40/132/82.5
				80/30/65.3
				80/60/53.3
				80/90/53.4
				80/132/42.6
2017 [64]	1-5	ECR-g/V/ 25/83 m	S/pH6.7	23/42/99.7
				23/125/98.5
				23/208/101.4
				40/42/97
				40/125/95.7
				40/208/94.7
				50/42/97.9
				50/125/96.3
				50/208/95.1
2017 [64]	1-6	ECR-g/V/ 12-25/82.5 m	S/pH6.7	23/42/104
				23/125/101.6
				23/208/98.2
				40/42/100.3
				40/125/100.9
				40/208/92.6
				50/42/96.5
				50/125/93.8
				50/208/89
2020 [34]	1-7	E-g/E/ 8/82.5 m	SW/8.1	23/30/100
				23/60/98
				23/90/98.9
				40/30/97.5
				40/60/93.3
				40/90/90
				60/30/93.9
				60/60/91.1
				60/90/82.6
2021 [65]	1-8	B/-/6/--	SW	28/15/88.1
				28/30/88.6
				28/90/88.6
				28/180/77.2
				28/360/66.8
				40/15/99.6
				40/30/77.8
				40/90/68
				40/180/62.6
				40/360/46.1
				60/15/87.3
				60/30/79.7
				60/90/53.8
				60/180/40.3
				60/360/29.6
2021 [65]	1-9	B/-/6/--	TW	28/15/99.7
				28/30/94.7
				28/90/94.6
				28/180/86.5
				28/360/81.2
				40/15/76.9
				40/30/92
				40/90/73.8
				40/180/79.8
				40/360/69.3
				60/15/86.5
				60/30/66.2
				60/90/62.2
				60/180/53.1
				60/360/41.9
2022 [60]	1-10	G/V/9.5 /82.3 v	DI	23/90/96.9
				23/180/98.7
				23/270/99.7
				23/360/97.8
				40/90/88.9
				40/180/80.3
				40/270/77
				40/360/74.3
				60/45/62.9
				60/90/55.2
				60/180/51.1
				60/270/48
				60/360/45.9
2022 [8]	1-11	ECR-g/V/ 10.2/76.3 m	SW/pH7.98	23/60/98.4
				23/120/98.1
				23/210/94.2
				23/365/92.3
				40/60/106.1
				40/120/98.6
				40/210/89.6
				40/365/79.2
				60/60/70.2
				60/120/60.3
				60/210/61.8
				60/365/55.4
2022 [8]	1-12	ECR-g/V/ 10.5/84.1m	SW/pH7.98	23/60/104
				23/120/102.2
				23/210/93.6
				23/365/96.9
				40/60/100.9
				40/120/100.7
				40/210/97.8
				40/365/92.5
				60/60/89.3
				60/120/94.8
				60/210/77.2
				60/365/74.6
2022 [8]	1-13	E-g/V/ 10.1/76.1 m	SW/ pH7.98	23/60/102.1
				23/120/100.9
				23/210/98
				23/365/93.9
				40/60/101.2
				40/120/98.8
				40/210/97
				40/365/94.6
				60/60/97.1
				60/120/85.4
				60/210/79
				60/365/78.7
2023 [41]	1-14	E-g/V/ 9.5/82.3 v	SW/ pH8.2	23/45/100
				23/90/99
				23/180/97.2
				23/270/96.1
				23/360/96.3
				40/45/96.5
				40/90/93.2
				40/180/83.8
				40/270/78.5
				40/360/75.4
				60/45/68.1
				60/90/55.7
				60/180/53.4
				60/270/50.1
				60/360/47.2
2020 [34]	1-15	E-g/E/ 8/82.5 m	SW/ pH8.1/ S20%	23/30/99.5
				23/60/96.3
				23/90/98.5
				40/30/98.4
				40/60/91.2
				40/90/87.6
				60/30/95.3
				60/60/87.5
				60/90/80.2
2020 [34]	1-16	E-g/E/ 8/82.5 m	SW/ pH8.1/ S40%	23/30/99.6
				23/60/96.9
				23/90/95.7
				40/30/93.4
				40/60/86.3
				40/90/82.3
				60/30/88
				60/60/81.7
				60/90/74.8

**Table A2:** Sub-database 2: bar exposure to alkaline solution.

Year	Group Number	FRP Type	Exposure Condition	*T*/*t*/TSR
2006 [46]	2-1	E-g/V/ 9.53/70 m	AS/13.6	20/60/81.8
				20/90/63.9
				20/120/54.7
				20/240/42.9
				40/60/69.1
				40/90/60.2
				40/120/50.1
				40/240/33
				60/60/52
				60/90/44
				60/120/38.1
				60/240/22.9
2006 [46]	2-2	E-g/V/ 9.53/70 m	AS/12.7	20/60/97.8
				20/70/96.6
				20/90/94.7
				20/120/91.6
				40/60/92.9
				40/70/91.9
				40/90/86
				40/120/86
				60/60/75.9
				60/70/73
				60/90/66
				60/120/59.1
2007 [66]	2-3	G/-/ 12.7/--	AS/12.6	20/30/98.9
				20/60/96.9
				20/90/94.2
				20/180/87
				20/239/87
				20/299/83.1
				40/29/98.5
				40/58/95.4
				40/88/92.6
				40/118/77.5
				40/237/76.5
				40/297/70.5
				60/30/87.9
				60/60/80.8
				60/90/74.9
				60/180/74
				60/239/72.8
				60/299/68.7
				80/4/91.3
				80/9/79.2
				80/13/77.4
				80/18/75.4
				80/24/73.3
				80/179/70.2
				80/237/69.2
				80/298/64.1
2008 [67]	2-4	G/-/12.7/--	AS/12.6	20/30/99.2
				20/60/97.1
				20/90/94.2
				20/180/87.1
				20/240/87.2
				20/300/83.1
				40/30/98.3
				40/60/95.4
				40/90/92.4
				40/180/85.4
				40/240/78
				40/300/70.1
				60/30/90.5
				60/60/85.6
				60/90/74.6
				60/180/73.5
				60/240/72.5
				60/300/68.5
				80/30/82
				80/60/80
				80/90/74.1
				80/180/70
				80/240/69.8
				80/300/64
2014 [9]	2-5	B/E/6/65 v	AS/ 12.9–13.1	25/21/99.1
				25/42/97.2
				25/63/94
				40/21/90.3
				40/42/88.9
				40/63/84.8
				55/21/82
				55/42/76.8
				55/63/68.5
2017 [35]	2-6	B/E/ 6/65 v	AS/13.4	32/21/97.5
				32/42/92.5
				32/63/92.7
				40/21/94.2
				40/42/88.7
				40/63/81.7
				48/21/93
				48/42/82.4
				48/63/59.1
				55/21/67.2
				55/42/37.5
				55/63/26
2017 [35]	2-7	B/E/ 6/65 v	AS/12.7	32/21/99.3
				32/42/93.8
				32/63/97.9
				40/21/95.3
				40/42/93
				40/63/90.2
				55/21/89.7
				55/42/81.1
				55/63/77.4
2017 [35]	2-8	E-g/E/ 6/62.8 v	AS/13.4	32/21/89.9
				32/42/91.2
				32/63/87.4
				40/21/85.3
				40/42/89.9
				40/63/90.8
				55/21/80.8
				55/42/68.7
				55/63/80.1
2017 [35]	2-9	E-g/E/ 6/62.8 v	AS/12.7	32/21/97.5
				32/42/95.2
				32/63/97.9
				40/21/90.6
				40/42/90.9
				40/63/94.1
				55/21/91.3
				55/42/92.6
				55/63/89.6
2018 [68]	2-10	G/P/ 12/78.8 m	AS/12.8	22/42/94.9
				22/125/94.6
				22/208/89.6
				40/42/87.7
				40/125/86.2
				40/208/82.3
				60/42/85.7
				60/125/84.7
				60/208/74.9
2018 [68]	2-11	G/V/ 12/83.9 m	AS/12.8	22/42/99.7
				22/125/97.2
				22/208/93.4
				40/42/99.3
				40/125/96.6
				40/208/87.1
				60/42/98.1
				60/125/95.7
				60/208/86.5
2018 [68]	2-12	G/E/ 12/79.4 m	AS/12.8	22/42/98.2
				22/125/98.1
				22/208/92.1
				40/42/97.2
				40/125/92.2
				40/208/87.2
				60/42/93.9
				60/125/91.3
				60/208/85.5
2018 [69]	2-13	ECR-g/V/ 8/88 m	AS/12.7	20/42/95
				20/83/94
				20/270/97
				40/42/91
				40/83/90
				40/270/84
				60/42/84
				60/83/73
				60/270/64
2018 [70]	2-14	ECR-g/V/ 10/80 m	AS/ 12.66–12.97	20/42/86.4
				20/90/85.5
				20/180/79.8
				20/365/82.6
				40/42/80.8
				40/90/80.7
				40/180/74.7
				40/365/77.5
				60/42/82.9
				60/90/75.2
				60/180/73.3
				60/365/75.7
2020 [34]	2-15	E-g/E/ 8/82.5 m	AS/13.4	23/30/95.2
				23/60/93.1
				23/90/90.3
				40/30/90.3
				40/60/88.4
				40/90/85.8
				60/30/82
				60/60/75.8
				60/90/67.8
2020 [71]	2-16	B/E/ 10.96/74.5 m	AS/12	20/90/95.7
				20/180/98.2
				20/270/95.8
				40/90/97.4
				40/180/95
				40/270/87.2
				60/90/91.5
				60/180/83
				60/270/71.5
2021 [72]	2-17	G/-/12 />85 v	AS/13	50/30/96.9
				50/90/95.4
				50/150/94.6
				60/30/94.7
				60/90/91.8
				60/150/89.7
				70/30/93.9
				70/90/86.6
				70/150/82.1
2021 [72]	2-18	G/-/ 16/>85 v	AS/13	50/30/98.7
				50/90/97.6
				50/150/97.2
				60/30/96.8
				60/90/94.4
				60/150/93
				70/30/95.2
				70/90/89.5
				70/150/86.4
2021 [65]	2-19	B/-/6/--	AS	28/15/89.6
				28/30/86.3
				28/91/76.4
				28/180/52.3
				28/360/43.7
				40/14/83.5
				40/30/83.1
				40/90/57.9
				40/180/54.5
				40/360/36.8
				60/15/85.9
				60/30/63.2
				60/90/42.9
				60/180/38.1
				60/360/18.4
2022 [60]	2-20	G/V/ 9.52/82.3 v	AS/11	23/45/98.7
				23/90/96.4
				23/180/96.1
				23/270/94.3
				23/360/92.7
				40/45/94
				40/90/88.3
				40/180/80.1
				40/270/74.6
				40/360/70
				60/45/61.7
				60/90/52.5
				60/180/49.2
				60/270/46.4
				60/360/44.2
2022 [60]	2-21	G/V/ 9.52/82.3 v	AS/12	23/45/97.7
				23/90/95.1
				23/180/96
				23/270/93.2
				23/360/91.1
				40/90/88.7
				40/180/79.1
				40/270/74.6
				40/360/67.2
				60/45/63.3
				60/90/50.9
				60/180/46.2
				60/270/44.3
				60/360/41
2022 [60]	2-22	G/V/ 9.52/82.3 v	AS/13	23/45/95.6
				23/90/92
				23/180/89
				23/270/84.5
				23/360/81.5
				40/45/92.2
				40/90/84.5
				40/180/68.7
				40/270/65.6
				40/360/61.9
				60/45/51.4
				60/90/39
				60/180/34
				60/270/30.9
				60/360/27.7
2022 [73]	2-23	C/E/ 6/66.8 v	AS/13.4	25/21/92.3
				25/42/91.4
				25/63/91.3
				25/84/90.6
				40/21/92.5
				40/42/85.8
				40/63/86.3
				40/84/81.4
				55/21/83
				55/42/77.6
				55/63/69.5
				55/84/64.1
2022 [74]	2-24	G/E/ 6/63 v	AS/13.4	25/21/98.4
				25/42/95.6
				25/63/92.2
				25/84/88.8
				40/21/89.5
				40/42/82.1
				40/63/69.5
				40/84/48.7
				55/21/49.2
				55/42/19.1
				55/63/4.8
2022 [75]	2-25	B/E/ 10/65 m	ASS	21/30/100.7
				21/60/97.6
				21/90/95.7
				40/30/96.1
				40/60/88.5
				40/90/80.6
				55/30/89
				55/60/49.1
				55/90/26.2
2022 [75]	2-26	H(B+C)/E /10/65 m	ASS	21/30/100.6
				21/60/96.3
				21/90/91.7
				40/30/95.6
				40/60/76.9
				40/90/76.1
				55/30/83.6
				55/60/61.2
				55/90/36.8
2022 [75]	2-27	H(B+C) /E/10/65 m	ASS	21/30/96.4
				21/60/99.3
				21/90/97.5
				40/30/98
				40/60/91
				40/90/83.3
				55/30/92
				55/60/70.1
				55/90/55.3
2023 [41]	2-28	E-g/V /9.52/82.3 v	AS/13.6	23/45/95.6
				23/90/86.3
				23/180/80.4
				23/270/77.7
				23/360/72.6
				40/45/87.4
				40/90/74
				40/180/59.6
				40/270/50.7
				40/360/44.4
				60/45/53
				60/90/39.7
				60/180/31.8
				60/270/30.9
				60/360/30.4
2023 [41]	2-29	E-g/V/ 9.52/82.3 v	ASS/13.6	23/45/90.4
				23/90/87.5
				23/180/79.6
				23/270/74.6
				23/360/70.3
				40/45/88.4
				40/90/74.8
				40/180/59.7
				40/270/49.5
				40/360/44.8
				60/45/50.2
				60/90/37.8
				60/180/36.6
				60/270/32.4
				60/360/31.9
2023 [14]	2-30	E-g/E/ 6/82.9 m	AS12.9	30/30/97.7
				30/60/98.6
				30/90/97
				30/180/95.7
				45/30/98.1
				45/60/98.1
				45/90/97.5
				45/180/90.7
				60/30/95.6
				60/60/92.2
				60/90/83
				60/180/59.3
2023 [14]	2-31	E-g/E/6/ 82.9 m	ASS12.9	30/30/100
				30/60/97.3
				30/90/96.9
				30/180/95.9
				45/30/99.1
				45/60/96.8
				45/90/95.4
				45/180/78.9
				60/30/94.7
				60/60/85.3
				60/90/73.6
				60/180/43.1
2023 [14]	2-32	B/E/6/ 83.2 m	AS12.9	30/30/96.6
				30/60/90.5
				30/90/91.4
				30/180/81.1
				45/30/92.4
				45/60/89.3
				45/90/83.6
				45/180/60.3
				60/30/69.9
				60/60/64
				60/90/52.5
2023 [14]	2-33	B/E/6/ 83.2 m	ASS12.9	30/30/94.5
				30/60/90.2
				30/90/91.5
				30/180/85.4
				45/30/93.5
				45/60/88
				45/90/79.2
				45/180/71.3
				60/30/81.5
				60/60/68.8
				60/90/67.9
				60/180/50.2
2024 [76]	2-34	SCG/E/ 8/60 V	AS12.9	20/21/96.5
				20/42/93.6
				20/63/91.8
				40/21/94.2
				40/42/88.3
				40/63/81.9
				60/21/92.4
				60/42/82.5
				60/63/77.2
2024 [77]	2-35	B/P/8/ 78.7 m	ASS9	25/7/96.2
				25/14/93.9
				25/28/92.5
				25/56/92
				25/112/88.1
				40/7/96.6
				40/14/95.5
				40/28/91
				40/56/90.3
				40/112/87.7
				60/7/93.5
				60/14/90.3
				60/28/85
				60/56/76.2
				60/112/74.4
2024 [77]	2-36	B/P/8/ 78.7 m	ASS10	25/7/98.4
				25/14/95.8
				25/28/92.3
				25/56/90.3
				25/112/88.5
				40/7/98.3
				40/14/93.7
				40/28/92.5
				40/56/89.3
				40/112/86.5
				60/7/89.6
				60/14/89.7
				60/28/89.1
				60/56/78.7
				60/112/73.8
2024 [77]	2-37	B/P/8/ 78.7 m	ASS11	25/7/98.4
				25/14/96.3
				25/28/95.1
				25/56/94.1
				25/112/93.6
				40/7/97.9
				40/14/94.9
				40/28/93.2
				40/56/91.3
				40/112/89.1
				60/7/96.4
				60/14/87.8
				60/28/80.7
				60/56/70.6
				60/112/70
2024 [77]	2-38	B/P/8/ 78.7 m	ASS12	25/7/97.2
				25/14/93.9
				25/28/91.8
				25/56/89
				25/112/88.1
				40/7/97.6
				40/14/93.5
				40/28/91.8
				40/56/87.2
				40/112/84.3
				60/7/94.8
				60/14/84.7
				60/28/79.8
				60/56/70.5
				60/112/68.4
2020 [34]	2-39	E-g/E /8/82.5 m	AS/13.4 /S0.20	23/30/93.8
				23/60/91.2
				23/90/86.4
				40/30/91.3
				40/60/85.1
				40/90/82.1
				60/30/83.2
				60/60/74.4
				60/90/65.2
2020 [34]	2-40	E-g/E /8/82.5 m	AS/13.4/ S0.40	23/30/91.1
				23/60/84.7
				23/90/78.2
				40/30/84.9
				40/60/78.6
				40/90/68
				60/30/76.2
				60/60/66.7
				60/90/53.2

**Table A3:** Sub-database 3: bars embedded into Mortar/Concrete.

Year	Group Number	FRP Bar	Exposure Condition	*T*/*t*/TSR
2002 [17]	3-1	G/V/ 9.5/--	M13.2/ AS8.5-9	30/10/83.7
				30/30/71.2
				30/60/66.5
				30/90/60.2
				30/180/53.7
				45/10/76.3
				45/30/66
				45/60/59.4
				45/90/58.6
				45/180/55.6
				57/10/76.5
				57/30/57.5
				57/60/56.4
				57/90/55
				57/180/39.9
2009 [29]	3-2	E-g/V/ 12.7/81.5 m	M12.15 /TW	23/120/89.1
				23/180/91
				23/240/90.6
				40/60/95.8
				40/120/84.5
				40/180/89.8
				40/240/89.8
				50/60/97.3
				50/120/91.4
				50/180/90.2
				50/240/84.4
2012 [10]	3-3	E-g/V/ 9.53/>70 m	C/TW	20/30/98.2
				20/90/92.5
				20/150/84.5
				20/210/82.6
				20/270/82.8
				40/30/87
				40/90/80.3
				40/150/69.8
				40/210/68.3
				40/270/65.2
				50/30/79.9
				50/90/66.8
				50/150/52.6
				50/210/50.4
				50/270/47.1
				60/30/78.2
				60/90/61.5
				60/150/47.9
				60/210/45.9
				60/270/44.8
2013 [33]	3-4	E-g/V/ 12.7/77.9 m	M12.15 /S	23/60/99.1
				23/120/98
				23/210/96.8
				23/365/92.1
				40/60/98.6
				40/120/96.4
				40/210/94.5
				40/365/90.4
				50/60/97
				50/120/95.6
				50/210/94.4
				50/365/89.1
2017 [59]	3-5	G/E/ 7.57/79 m	SC/TW	20/150/98.1
				20/300/98.9
				20/450/98.6
				40/150/91.9
				40/300/94.7
				40/450/92
				60/150/90
				60/300/93.6
				60/450/85.4
2017 [59]	3-6	G/E/ 8.29/75 m	SC/TW	20/150/81.1
				20/300/78.8
				20/450/72.7
				40/150/76.7
				40/300/65.9
				40/450/68
				60/150/70.8
				60/300/50.1
				60/450/53.1
2020 [71]	3-7	B/E/ 10.96/74.5 m	C12.5/TW	20/90/94.4
				20/180/93.8
				20/270/90.7
				40/90/93.6
				40/180/90.2
				40/270/90.4
				60/90/88
				60/180/88.4
				60/270/85.1
2021 [65]	3-8	B/-/6/--	SSM/AS /10 mm cover	28/15/77.3
				28/30/66.6
				28/90/52.7
				28/180/48.9
				28/360/42.8
				40/15/69.4
				40/30/62.7
				40/90/48.2
				40/180/42.3
				40/360/31.2
				60/15/68.6
				60/30/61.3
				60/90/41.3
				60/180/23.5
				60/360/0
2021 [65]	3-9	B/-/6/--	SSM/SW/ 10 mm cover	28/15/83.6
				28/30/72.7
				28/90/66.9
				28/180/66.7
				28/360/63.6
				40/15/69.4
				40/30/70.8
				40/90/58.4
				40/180/56.9
				40/360/54.8
				60/15/66.7
				60/30/63.6
				60/90/55.4
				60/180/50.6
				60/360/38
2021 [65]	3-10	B/-/6/--	SSM/TW/ 10 mm cover	28/15/84
				28/30/75.5
				28/90/63.7
				28/180/64.3
				28/360/56.7
				40/15/69.4
				40/30/62
				40/90/53.1
				40/180/52.2
				40/360/46.3
				60/15/64.3
				60/30/57.7
				60/90/47.9
				60/180/40.1
				60/360/35.6
2021 [65]	3-11	B/-/6/--	SSM/AS/ 20 mm cover	28/15/73.6
				28/30/61.4
				28/90/39.8
				28/180/31.9
				28/360/21.5
				40/15/62.3
				40/30/50.6
				40/90/28.6
				40/180/24.6
				40/360/0
				60/15/58.7
				60/30/46.9
				60/90/18.5
				60/180/0
2021 [65]	3-12	B/-/6/--	SSM/SW/ 20 mm cover	28/15/71.5
				28/30/61.1
				28/90/47.4
				28/180/39.7
				28/360/38.1
				40/15/58.8
				40/30/51.4
				40/90/46.2
				40/180/33.4
				40/360/27.7
				60/15/61.9
				60/30/45.5
				60/90/29.6
				60/180/25.1
				60/360/0
2021 [65]	3-13	B/-/6/--	SSM/TW/ 20 mm cover	28/15/69.9
				28/30/61.5
				28/90/41.4
				28/180/35.3
				28/360/34.7
				40/15/60.2
				40/30/52
				40/90/34
				40/180/22.8
				40/360/21.5
				60/15/53.9
				60/30/43.9
				60/90/25.1
				60/180/22.4
				60/360/0
2022 [62]	3-14	E-g/V /9.5/82.3 v	M11.3/water	23/90/93.6
				23/180/94.3
				23/270/93.6
				23/360/92.9
				40/90/85.9
				40/180/71.9
				40/270/71.9
				40/360/68.2
				60/45/64.4
				60/90/55.2
				60/180/46.4
				60/270/48.4
				60/360/44.9
2022 [62]	3-15	E-g/V/ 9.5/82.3 v	M11.8/water	23/45/94.7
				23/90/95.3
				23/180/91.5
				23/270/91.9
				23/360/90
				40/45/92.9
				40/90/86
				40/180/72.1
				40/270/67.4
				40/360/62
				60/45/64.3
				60/90/54.9
				60/180/46.6
				60/270/44.4
				60/360/44.6
2022 [62]	3-16	E-g/V/ 9.5/82.3 v	M12.8/ water	23/45/90.8
				23/90/90.1
				23/180/89
				23/270/85.7
				23/360/83.9
				40/45/77.9
				40/90/73.6
				40/180/68.9
				40/270/61.9
				40/360/58.3
				60/45/55.4
				60/90/46.6
				60/180/45.1
				60/270/43.3
				60/360/42.2
2012 [10]	3-17	E-g/V/ 9.53/>70 m	C/TW/ stressed	20/90/89.1
				20/120/84.1
				20/170/84.1
				20/210/79.9
				40/90/83.9
				40/120/80.1
				40/210/70
				60/90/63
				60/120/60
				60/170/48.1
				60/210/47.1
2018 [69]	3-18	ECR-g/V /8/88 m	C/TW /s3000με	20/42/96
				20/83/92
				20/270/89
				40/42/87
				40/83/87
				40/270/76
				60/42/82
				60/83/74
				60/270/59

## Appendix B. Model Parameter of Different Models Based on Accelerated Test Data

**Table A4:** Exponential model parameter values based on the accelerated test data.

Group Number	*T*_1_		*T*_2_		*T*_3_		*T*_4_		*E_a_*/*R*
	*τ*	*R* ^2^	*τ*	*R* ^2^	*τ*	*R* ^2^	*τ*	*R* ^2^	
1-1	616.40	0.33	550.76	0.57	384.60	0.48	-	-	918.20
1-2	543.26	0.24	488.35	0.83	122.31	0.86	-	-	3019.10
1-3	571.73	0.45	629.90	0.34	449.27	0.66	-	-	-
1-4	522.66	0.87	461.40	0.42	123.09	0.83	-	-	2918.00
1-5	−65,568.75	0.02	3325.36	0.75	3781.01	0.89	-	-	-
1-6	818,230.43	−0.20	4222.57	0.51	1791.44	0.98	-	-	22,592.00
1-7	5648.37	0.49	876.01	0.99	513.82	0.96	-	-	6364.80
1-8	791.39	0.73	376.52	0.82	198.13	0.91	-	-	4262.60
1-9	1567.38	0.92	766.53	−0.08	281.62	0.60	-	-	-
1-10	17,648.83	−0.24	1046.57	0.92	299.57	0.41	-	-	-
1-11	4329.73	0.95	1915.80	0.73	420.60	0.47	-	-	6230.20
1-12	9849.09	0.25	6265.42	0.70	1106.23	0.81	-	-	5854.70
1-13	8035.92	0.59	7198.77	0.83	1203.57	0.81	-	-	5095.40
1-14	8050.71	0.91	1175.77	0.98	322.84	0.48	-	-	8562.60
1-15	3343.91	0.37	706.40	0.93	433.72	0.98	-	-	5424.30
1-16	2095.36	0.91	441.71	0.99	296.50	0.98	-	-	5190.40
2-1	239.44	0.95	185.66	0.98	117.79	0.95	-	-	1719.20
2-2	1627.94	0.89	740.08	0.94	222.25	1.00	-	-	4833.70
2-3	1575.36	0.97	816.40	0.88	633.17	0.49	553.28	−0.82	1786.20
2-4	1594.33	0.97	957.42	0.97	643.64	0.67	545.49	0.48	1888.70
2-5	1175.63	0.97	349.48	0.87	154.93	0.94	-	-	6625.00
2-6	717.30	0.89	325.99	0.99	153.94	0.87	46.47	0.99	11,559.00
2-7	1482.34	0.29	582.72	0.97	224.33	0.97	-	-	8032.40
2-8	418.14	0.67	434.77	−0.05	172.94	0.47	-	-	4120.70
2-9	1562.92	0.19	613.17	−0.01	506.60	0.63	-	-	-
2-10	1885.72	0.82	917.43	0.60	683.30	0.77	-	-	2629.60
2-11	3397.22	0.93	1855.86	0.84	1685.23	0.89	-	-	1817.50
2-12	3016.17	0.82	1529.36	1.00	1295.50	0.92	-	-	2190.20
2-13	5172.94	−0.97	1322.57	0.57	480.96	0.68	-	-	5810.10
2-14	1259.77	−0.12	919.51	−0.20	825.43	−0.11	-	-	-
2-15	839.65	0.96	512.73	0.83	214.68	0.95	-	-	3643.70
2-16	6065.97	0.26	2362.60	0.88	869.00	0.98	-	-	4734.80
2-17	2317.07	0.73	1216.09	0.82	701.74	0.96	-	-	6626.10
2-18	4593.51	0.83	1836.32	0.86	935.10	0.95	-	-	8836.10
2-19	347.16	0.92	272.51	0.82	135.14	0.87	-	-	3013.90
2-20	4510.56	0.92	922.34	0.97	279.45	0.41	-	-	7407.50
2-21	3714.65	0.84	874.52	0.99	257.11	0.52	-	-	7115.50
2-22	1621.99	0.95	634.09	0.94	147.16	0.64	-	-	6408.70
2-23	668.33	0.44	377.17	0.89	173.09	0.95	-	-	4391.70
2-24	765.94	0.97	150.19	0.91	26.46	0.99	-	-	10,961.00
2-25	2449.37	0.73	456.13	0.96	88.68	0.88	-	-	9337.10
2-26	1291.80	0.77	304.14	0.88	109.67	0.95	-	-	7002.60
2-27	3305.12	−0.06	563.94	0.91	170.28	0.94	-	-	8429.90
2-28	1007.87	0.92	387.65	0.97	149.24	0.64	-	-	5092.30
2-29	907.04	0.92	387.98	0.98	156.51	0.51	-	-	-
2-30	3674.87	0.73	2106.79	0.90	407.92	0.93	-	-	7337.40
2-31	3731.88	0.81	955.49	0.84	259.48	0.94	-	-	8971.40
2-32	849.06	0.95	402.05	0.96	126.66	0.92	-	-	6378.80
2-33	997.47	0.78	481.54	0.96	223.46	0.91	-	-	5030.30
2-34	690.42	0.98	324.95	1.00	236.83	0.99	-	-	2631.60
2-35	735.56	0.44	679.22	0.55	286.04	0.69	-	-	2738.80
2-36	729.74	0.68	631.35	0.70	300.09	0.66	-	-	2565.10
2-37	1277.38	0.31	782.53	0.61	227.42	0.71	-	-	4947.00
2-38	669.90	0.47	535.47	0.75	214.14	0.66	-	-	3286.00
2-39	609.99	0.98	413.41	0.96	201.76	0.98	-	-	2956.30
2-40	360.75	1.00	231.86	0.98	139.23	0.98	-	-	2539.50
3-1	196.99	0.58	178.55	0.15	128.96	0.53	-	-	-
3-2	1895.24	0.61	1573.09	0.42	1512.37	0.96	-	-	827.10
3-3	1185.17	0.92	522.43	0.85	282.19	0.88	246.77	0.86	4027.30
3-4	4951.71	0.95	3619.66	1.00	3169.43	0.96	-	-	1597.60
3-5	25,122.09	0.01	4716.34	0.38	2955.35	0.68	-	-	5280.80
3-6	1245.47	0.84	886.00	0.80	542.95	0.89	-	-	2016.60
3-7	2623.14	0.88	2162.93	0.78	1443.90	0.69	-	-	1444.70
3-8	239.67	0.38	165.58	0.50	88.59	0.86	-	-	-
3-9	509.52	−0.19	343.74	−0.31	227.24	0.20	-	-	-
3-10	428.06	0.25	255.83	−0.12	156.76	0.22	-	-	-
3-11	109.28	0.79	56.49	0.80	38.71	0.95	-	-	-
3-12	169.05	0.37	107.16	0.35	51.72	0.76	-	-	-
3-13	131.92	0.48	63.84	0.71	38.98	0.77	-	-	-
3-14	3967.09	0.36	783.26	0.87	287.03	0.47	-	-	-
3-15	2887.96	0.60	674.78	0.97	275.28	0.52	-	-	-
3-16	1721.84	0.52	529.89	0.66	239.02	0.29	-	-	-
3-17	880.21	0.93	562.65	0.99	239.64	0.97	-	-	3151.20
3-18	1994.80	0.68	854.72	0.68	423.03	0.80	-	-	3788.90

**Table A5:** Modified exponential model parameter values based on the accelerated test data.

Group Number	*T*_1_			*T*_2_			*T*_3_			*T*_4_			*E_a_*/*R*
	*n*	*τ*	*R* ^2^	*n*	*τ*	*R* ^2^	*n*	*τ*	*R* ^2^	*n*	*τ*	*R* ^2^	
1-1	0.247	19.81	0.72	0.282	20.90	0.97	0.221	11.26	0.93	-	-	-	-
1-2	0.273	19.60	0.53	0.617	84.78	0.90	0.469	11.75	1.00	-	-	-	7911.60
1-3	0.319	25.46	0.76	0.185	15.34	0.88	0.376	26.19	0.93	-	-	-	-
1-4	0.659	109.45	0.93	0.255	15.54	0.82	0.432	10.03	0.99	-	-	-	-
1-5	40.222	−1.81 × 10^96^	0.45	0.370	132.64	1.00	0.540	358.07	1.00	-	-	-	-
1-6	−1.883	−0.02	0.71	3.241	452,749,669.94	0.88	0.851	833.41	0.98	-	-	-	-
1-7	0.790	2294.36	0.50	1.200	2067.55	1.00	1.193	1177.49	0.97	-	-	-	9231.23
1-8	0.495	49.50	0.91	0.618	48.47	0.93	0.663	36.62	0.99	-	-	-	6069.20
1-9	0.751	388.79	0.95	0.208	10.36	0.71	0.427	14.14	0.95	-	-	-	-
1-10	−0.834	0.79	0.62	0.613	119.93	0.99	0.237	5.11	1.00	-	-	-	-
1-11	0.950	3257.82	0.96	2.007	573,353.60	0.88	0.243	7.14	0.98	-	-	-	-
1-12	1.552	218,128.83	0.29	2.708	111,006,653.30	0.95	0.785	334.80	0.83	-	-	-	-
1-13	2.709	1.38 × 10^8^	0.84	1.552	165,027.36	0.90	0.677	199.30	0.87	-	-	-	-
1-14	0.918	5072.04	0.92	0.918	744.89	0.99	0.281	6.85	0.99	-	-	-	12,403.73
1-15	0.515	417.30	0.44	1.423	4363.01	0.97	1.331	1794.53	1.00	-	-	-	-
1-16	1.422	12,941.55	0.95	0.909	299.33	0.99	0.759	106.29	1.00	-	-	-	2810.10
2-1	0.844	111.41	0.96	0.793	68.05	1.00	0.588	17.16	1.00	-	-	-	2923.23
2-2	1.814	66,834.98	1.00	1.002	747.09	0.94	0.949	176.83	1.00	-	-	-	-
2-3	0.973	1363.23	0.97	0.943	602.83	0.89	0.362	21.30	0.96	0.194	7.15	0.90	-
2-4	0.995	1547.83	0.97	1.335	5899.18	0.99	0.470	38.05	0.94	0.343	16.75	0.99	-
2-5	1.848	34,258.84	1.00	0.453	41.51	0.98	0.604	33.45	0.99	-	-	-	-
2-6	0.784	307.50	0.90	1.156	602.63	1.00	2.169	15,515.66	0.99	1.115	71.07	1.00	13,212.75
2-7	0.462	178.70	0.36	0.716	191.19	1.00	0.746	83.31	0.99	-	-	-	18,636.00
2-8	0.203	18.94	0.94	−0.486	1.45	1.00	0.107	5.52	0.82	-	-	-	-
2-9	0.008	32.40	0.63	−0.342	3.43	0.95	0.153	18.94	0.94	-	-	-	-
2-10	0.538	177.78	0.90	0.242	19.67	0.98	0.413	34.73	0.93	-	-	-	-
2-11	1.764	178,860.03	1.00	2.624	8,805,456.43	1.00	2.102	521,042.53	0.98	-	-	-	-
2-12	2.458	6.17 × 10^6^	0.92	0.997	1504.48	1.00	0.635	199.88	0.97	-	-	-	-
2-13	−0.248	6.83	0.89	0.357	42.95	0.99	0.437	25.11	0.98	-	-	-	-
2-14	0.152	11.69	0.95	0.113	7.13	0.97	0.153	7.96	0.95	-	-	-	-
2-15	0.689	222.47	1.00	0.373	35.80	1.00	0.625	44.00	1.00	-	-	-	-
2-16	−0.193	10.82	0.69	2.010	573,531.22	0.98	1.279	3884.13	1.00	-	-	-	-
2-17	0.351	104.12	1.00	0.432	80.57	1.00	0.694	162.23	1.00	-	-	-	25,753.50
2-18	0.465	353.21	0.99	0.494	162.80	1.00	0.654	178.57	1.00	-	-	-	22,926.00
2-19	0.707	72.68	0.97	0.563	27.83	0.98	0.606	20.98	0.97	-	-	-	3928.63
2-20	0.705	859.69	0.96	0.780	271.49	1.00	0.233	4.76	1.00	-	-	-	14,566.50
2-21	0.616	432.08	0.92	0.828	334.36	1.00	0.288	5.98	0.99	-	-	-	14,132.25
2-22	0.706	313.50	1.00	0.750	159.86	0.97	0.296	4.37	1.00	-	-	-	13,145.75
2-23	0.136	18.76	1.00	0.611	74.87	0.96	0.671	44.68	0.99	-	-	-	35,052.50
2-24	1.403	4171.22	1.00	1.699	2760.33	0.98	1.276	69.22	1.00	-	-	-	8733.58
2-25	2.320	748,329.63	0.90	1.478	3566.44	1.00	2.013	5988.89	0.99	-	-	-	4324.53
2-26	2.529	988,864.76	0.97	1.134	538.89	0.89	1.609	1418.10	1.00	-	-	-	-
2-27	0.000	43.36	0.48	1.798	17,662.27	1.00	1.589	2078.15	0.99	-	-	-	-
2-28	0.699	188.49	0.97	0.786	120.73	0.99	0.299	4.49	0.99	-	-	-	11,178.20
2-29	0.643	124.94	1.00	0.815	141.50	0.99	0.225	3.16	0.99	-	-	-	13,307.23
2-30	0.545	399.92	0.87	1.535	30,145.36	0.96	1.545	5831.41	1.00	-	-	-	14,619.50
2-31	0.751	1096.93	0.84	2.109	241,695.10	1.00	1.498	2825.28	1.00	-	-	-	11,623.25
2-32	0.902	526.37	0.96	1.309	1811.32	0.99	0.539	18.44	0.99	-	-	-	7396.63
2-33	0.529	101.32	0.96	0.826	207.88	0.98	0.646	42.38	0.99	-	-	-	8879.85
2-34	0.772	281.61	1.00	1.116	511.59	1.00	1.004	241.01	0.99	-	-	-	3916.43
2-35	0.373	47.56	0.98	0.430	55.71	0.96	0.502	33.13	0.96	-	-	-	7924.33
2-36	0.504	82.05	0.94	0.508	72.59	0.96	0.474	30.93	0.95	-	-	-	4663.40
2-37	0.354	74.40	0.94	0.452	70.48	0.97	0.546	32.25	0.92	-	-	-	13,974.00
2-38	0.405	49.35	0.95	0.533	68.98	0.97	0.501	25.20	0.94	-	-	-	7835.03
2-39	0.808	268.25	0.99	0.680	105.70	1.00	0.783	80.56	1.00	-	-	-	3619.95
2-40	0.898	233.39	1.00	0.820	107.97	0.99	0.789	57.35	0.99	-	-	-	2875.10
3-1	0.402	12.52	0.99	0.252	5.90	0.99	0.365	7.60	0.97	-	-	-	-
3-2	−0.260	2.55	0.99	0.343	51.23	0.65	1.146	3247.50	0.97	-	-	-	-
3-3	0.794	396.57	0.94	0.533	45.03	0.99	0.561	29.12	0.99	0.537	22.86	0.99	5742.90
3-4	1.402	48,538.55	0.99	1.010	3829.49	1.00	0.846	1333.86	0.97	-	-	-	-
3-5	0.000	67.37	0.83	0.000	13.58	0.88	0.415	95.76	0.77	-	-	-	-
3-6	0.386	34.63	0.99	0.344	19.62	0.97	0.547	39.84	0.96	-	-	-	4296.90
3-7	0.507	186.16	0.97	0.365	72.20	0.98	0.207	21.05	0.98	-	-	-	-
3-8	0.339	8.16	0.98	0.358	7.15	1.00	0.555	13.00	1.00	-	-	-	-
3-9	0.225	7.79	0.95	0.185	4.72	0.98	0.272	5.53	0.99	-	-	-	-
3-10	0.315	10.89	0.97	0.214	4.49	0.99	0.270	4.60	1.00	-	-	-	-
3-11	0.491	10.98	0.99	0.459	7.03	0.99	0.656	11.67	1.00	-	-	-	-
3-12	0.323	6.24	0.98	0.278	4.05	0.99	0.417	5.79	0.99	-	-	-	-
3-13	0.350	6.40	0.98	0.382	5.42	0.99	0.379	4.41	0.99	-	-	-	3684.25
3-14	0.074	22.45	0.98	0.561	67.86	0.96	0.269	5.89	0.99	-	-	-	-
3-15	0.374	88.04	0.95	0.816	243.11	0.99	0.291	6.39	0.99	-	-	-	16,718.75
3-16	0.315	38.50	0.97	0.385	18.30	0.99	0.164	2.97	0.99	-	-	-	20,124.25
3-17	0.645	144.75	0.97	0.838	248.30	1.00	0.646	40.64	0.99	-	-	-	4797.23
3-18	0.451	104.62	0.96	0.426	40.50	0.98	0.508	32.42	1.00	-	-	-	7901.45

**Table A6:** Single logarithmic model parameter values based on the accelerated test data.

Group Number	*T*_1_			*T*_2_			*T*_3_			*T*_4_			*E_a_*/*R*
	*a*	*b*	*R* ^2^	*a*	*b*	*R* ^2^	*a*	*b*	*R* ^2^	*a*	*b*	*R* ^2^	
1-1	−8.25	101.70	0.21	−9.08	101.96	0.79	−9.70	97.46	0.47	-	-	-	4816.10
1-2	−11.33	105.76	0.14	−20.40	122.08	0.80	−35.16	118.11	1.00	-	-	-	-
1-3	−11.27	106.53	0.36	−5.87	97.44	0.29	−14.44	109.21	0.73	-	-	-	-
1-4	−20.79	123.82	0.90	−10.38	101.72	0.31	−32.30	112.85	0.92	-	-	-	-
1-5	1.61	96.63	0.16	−3.25	102.34	0.98	−3.93	104.35	0.99	-	-	-	-
1-6	−7.84	117.05	0.91	−9.05	116.13	0.49	−10.06	113.33	0.88	-	-	-	14,669.50
1-7	−2.65	103.55	0.42	−15.48	120.46	0.99	−22.00	127.39	0.82	-	-	-	-
1-8	−14.72	109.59	0.72	−34.12	135.03	0.94	−44.22	141.54	0.99	-	-	-	-
1-9	−12.46	114.84	0.89	−8.19	93.78	0.29	−28.49	115.68	0.93	-	-	-	-
1-10	2.59	92.35	0.31	−24.11	135.58	0.99	−18.18	92.07	0.99	-	-	-	-
1-11	−8.33	113.94	0.89	−34.37	168.46	0.98	−16.67	98.35	0.83	-	-	-	23,710.00
1-12	−11.53	124.37	0.66	−10.53	120.97	0.82	−23.07	134.42	0.64	-	-	-	-
1-13	−10.39	121.43	0.92	−8.27	115.99	1.00	−24.26	138.10	0.89	-	-	-	-
1-14	−4.63	107.78	0.97	−24.55	138.75	0.97	−21.10	100.70	0.92	-	-	-	46,060.25
1-15	−3.10	103.50	0.21	−22.75	131.89	1.00	−31.04	141.56	0.98	-	-	-	-
1-16	−8.31	111.80	0.99	−23.39	127.96	1.00	−26.82	128.05	0.97	-	-	-	-
2-1	−62.75	189.31	0.94	−61.14	178.32	1.00	−48.58	138.75	1.00	-	-	-	2573.35
2-2	−20.46	134.35	0.99	−25.69	138.40	0.84	−57.44	178.47	1.00	-	-	-	7894.43
2-3	−16.06	124.14	0.95	−28.93	143.48	0.88	−16.92	111.23	0.92	−10.31	90.97	0.82	-
2-4	−16.26	124.71	0.95	−26.53	141.09	0.88	−20.99	120.65	0.90	−17.02	108.28	0.94	-
2-5	−10.22	112.93	0.91	−10.79	105.06	0.83	−27.11	118.64	0.92	-	-	-	20,257.50
2-6	−10.77	111.26	0.84	−25.35	128.29	0.95	−67.19	184.44	0.87	−87.68	182.24	0.99	-
2-7	−4.59	104.25	0.15	−10.36	109.22	0.96	−26.08	123.98	0.99	-	-	-	-
2-8	−4.21	96.16	0.28	11.93	69.80	0.95	−5.64	85.45	0.04	-	-	-	-
2-9	−0.07	96.98	0.00	6.65	81.34	0.68	−2.71	95.46	0.19	-	-	-	-
2-10	−6.50	106.08	0.60	−7.00	99.47	0.80	−13.36	108.64	0.63	-	-	-	-
2-11	−8.41	113.67	0.89	−15.49	125.49	0.75	−14.81	123.21	0.74	-	-	-	-
2-12	−7.32	110.85	0.57	−13.72	119.83	0.96	−11.04	112.45	0.84	-	-	-	-
2-13	2.83	89.71	0.58	−8.96	106.16	0.94	−23.90	121.25	0.96	-	-	-	-
2-14	−5.44	95.01	0.54	−5.08	89.08	0.49	−7.66	92.85	0.54	-	-	-	-
2-15	−9.92	110.08	0.95	−9.10	103.96	0.94	−28.77	125.17	0.95	-	-	-	-
2-16	1.20	93.93	0.04	−20.06	137.60	0.82	−40.53	171.72	0.95	-	-	-	-
2-17	−3.27	101.74	1.00	−6.98	105.12	0.99	−16.63	118.62	0.99	-	-	-	-
2-18	−2.17	101.89	1.00	−5.37	104.77	1.00	−12.49	113.71	1.00	-	-	-	-
2-19	−35.23	136.09	0.92	−34.34	127.71	0.94	−45.42	135.36	0.97	-	-	-	3379.55
2-20	−5.96	108.57	0.91	−26.50	138.91	0.99	−18.32	90.56	0.96	-	-	-	36,204.25
2-21	−6.17	108.00	0.79	−34.07	155.75	0.98	−22.96	98.97	0.94	-	-	-	33,552.50
2-22	−15.06	121.20	0.96	−35.25	151.07	0.98	−24.80	90.44	0.96	-	-	-	16,620.75
2-23	−2.54	95.61	0.93	−16.64	114.25	0.89	−31.42	125.94	0.95	-	-	-	-
2-24	−15.49	119.56	0.94	−62.75	177.07	0.84	−93.95	172.95	1.00	-	-	-	17,939.00
2-25	−10.56	116.35	1.00	−31.63	143.30	0.97	−131.77	283.62	1.00	-	-	-	-
2-26	−18.21	127.81	0.97	−43.09	157.72	0.89	−95.49	226.33	0.97	-	-	-	-
2-27	3.19	92.18	0.28	−30.13	143.10	0.97	−76.63	205.51	1.00	-	-	-	-
2-28	−23.90	134.40	0.98	−47.66	166.65	1.00	−25.01	91.44	0.91	-	-	-	11,347.00
2-29	−22.63	129.58	0.96	−49.20	170.22	1.00	−18.95	78.91	0.88	-	-	-	13,047.75
2-30	−2.89	102.64	0.59	−9.21	113.29	0.69	−46.92	170.09	0.87	-	-	-	-
2-31	−5.12	107.09	0.91	−25.08	139.36	0.78	−66.32	197.94	0.93	-	-	-	-
2-32	−18.70	124.80	0.89	−40.82	157.58	0.84	−34.65	122.31	0.89	-	-	-	11,997.93
2-33	−10.77	110.49	0.86	−29.57	138.19	0.97	−38.51	138.97	0.95	-	-	-	-
2-34	−9.83	109.51	1.00	−25.11	127.85	0.97	−31.97	134.59	1.00	-	-	-	-
2-35	−6.01	101.24	0.93	−7.64	103.28	0.95	−17.37	109.02	0.96	-	-	-	-
2-36	−8.40	105.22	0.98	−9.30	105.52	0.98	−14.15	104.66	0.82	-	-	-	-
2-37	−3.92	101.17	0.94	−7.04	103.47	0.99	−23.25	114.75	0.96	-	-	-	-
2-38	−7.67	103.10	0.97	−10.93	106.70	0.99	−22.26	111.85	0.96	-	-	-	-
2-39	−14.77	116.11	0.90	−19.42	119.88	1.00	−36.84	138.27	0.97	-	-	-	-
2-40	−26.40	130.54	0.98	−33.73	135.76	0.91	−46.43	146.01	0.94	-	-	-	4527.58
3-1	−23.71	107.21	0.99	−16.80	91.62	0.96	−25.87	100.83	0.91	-	-	-	-
3-2	5.43	78.09	0.67	−8.91	108.92	0.25	−19.58	132.41	0.93	-	-	-	-
3-3	−17.90	125.21	0.94	−23.69	123.21	0.95	−35.91	133.79	0.97	−37.26	132.85	0.97	9142.45
3-4	−8.35	114.76	0.84	−10.16	117.18	0.95	−9.35	114.44	0.83	-	-	-	-
3-5	1.33	95.30	0.63	1.21	89.96	0.03	−7.30	107.48	0.19	-	-	-	-
3-6	−16.52	117.79	0.85	−20.16	119.31	0.72	−40.48	156.61	0.76	-	-	-	-
3-7	−7.03	108.52	0.74	−7.10	107.09	0.82	−5.35	99.02	0.50	-	-	-	-
3-8	−24.62	104.01	0.98	−27.23	102.03	0.99	−42.10	120.99	0.98	-	-	-	-
3-9	−13.12	95.40	0.86	−12.50	85.59	0.89	−19.75	92.12	0.94	-	-	-	-
3-10	−18.77	104.19	0.95	−15.82	86.36	0.97	−21.27	89.24	1.00	-	-	-	-
3-11	−37.96	117.10	0.99	−36.98	105.06	0.97	−52.52	122.16	0.99	-	-	-	-
3-12	−25.07	98.76	0.96	−22.53	86.01	0.96	−34.11	99.14	0.96	-	-	-	-
3-13	−27.64	100.54	0.94	−30.55	95.69	0.97	−30.93	89.43	0.97	-	-	-	3499.00
3-14	−0.99	95.89	0.20	−28.31	139.56	0.89	−21.00	97.43	0.91	-	-	-	-
3-15	−5.50	104.59	0.81	−35.16	152.38	0.99	−22.77	100.38	0.95	-	-	-	-
3-16	−7.38	103.93	0.84	−21.55	114.90	0.95	−13.46	75.71	0.88	-	-	-	27,436.25
3-17	−21.25	129.92	0.86	−38.25	159.03	1.00	−48.49	158.67	0.94	-	-	-	6315.20
3-18	−8.33	108.92	0.95	−14.40	112.00	0.87	−28.45	128.30	1.00	-	-	-	-

**Table A7:** Double logarithmic model parameter values based on the accelerated test data.

Group Number	*T*_1_			*T*_2_			*T*_3_			*T*_4_			*E_a_*/*R*
	*a*	*b*	*R* ^2^	*a*	*b*	*R* ^2^	*a*	*b*	*R* ^2^	*a*	*b*	*R* ^2^	
1-1	−0.042	2.015	0.21	−0.046	2.016	0.80	−0.054	1.999	0.48	-	-	-	-
1-2	−0.061	2.041	0.15	−0.104	2.119	0.81	−0.277	2.233	1.00	-	-	-	-
1-3	−0.059	2.041	0.37	−0.030	1.993	0.30	−0.077	2.058	0.75	-	-	-	-
1-4	−0.106	2.126	0.91	−0.056	2.020	0.32	−0.253	2.190	0.91	-	-	-	-
1-5	0.007	1.985	0.16	−0.015	2.011	0.98	−0.018	2.020	0.99	-	-	-	-
1-6	−0.033	2.072	0.90	−0.039	2.070	0.48	−0.046	2.062	0.87	-	-	-	-
1-7	−0.012	2.016	0.42	−0.071	2.095	0.99	−0.105	2.132	0.81	-	-	-	-
1-8	−0.075	2.052	0.69	−0.209	2.231	0.95	−0.321	2.341	0.97	-	-	-	-
1-9	−0.058	2.070	0.88	−0.044	1.976	0.28	−0.200	2.157	0.93	-	-	-	-
1-10	0.011	1.966	0.31	−0.129	2.200	0.99	−0.148	2.039	0.99	-	-	-	-
1-11	−0.038	2.063	0.89	−0.157	2.311	0.97	−0.117	2.047	0.84	-	-	-	-
1-12	−0.051	2.107	0.66	−0.046	2.091	0.81	−0.116	2.175	0.63	-	-	-	-
1-13	−0.045	2.093	0.91	−0.037	2.071	0.99	−0.126	2.204	0.91	-	-	-	-
1-14	−0.021	2.035	0.97	−0.121	2.193	0.96	−0.166	2.096	0.94	-	-	-	-
1-15	−0.014	2.016	0.21	−0.106	2.150	1.00	−0.151	2.205	0.98	-	-	-	-
1-16	−0.037	2.053	1.00	−0.115	2.141	1.00	−0.141	2.154	0.96	-	-	-	-
2-1	−0.492	2.777	0.98	−0.517	2.770	0.98	−0.553	2.712	0.98	-	-	-	1890.375
2-2	−0.094	2.158	0.99	−0.127	2.192	0.84	−0.368	2.539	0.99	-	-	-	-
2-3	−0.075	2.113	0.94	−0.144	2.217	0.86	−0.096	2.079	0.93	−0.062	1.969	0.842	-
2-4	−0.076	2.116	0.95	−0.127	2.196	0.86	−0.117	2.127	0.91	−0.099	2.066	0.930	-
2-5	−0.046	2.058	0.91	−0.053	2.028	0.82	−0.152	2.119	0.91	-	-	-	-
2-6	−0.050	2.053	0.85	−0.123	2.139	0.94	−0.351	2.443	0.84	−0.855	2.959	1.000	-
2-7	−0.021	2.020	0.15	−0.048	2.044	0.96	−0.136	2.132	1.00	-	-	-	-
2-8	−0.020	1.984	0.27	0.058	1.855	0.95	−0.033	1.937	0.04	-	-	-	-
2-9	0.000	1.987	0.00	0.032	1.913	0.69	−0.013	1.980	0.19	-	-	-	-
2-10	−0.030	2.029	0.60	−0.035	2.002	0.79	−0.069	2.051	0.62	-	-	-	-
2-11	−0.037	2.061	0.89	−0.070	2.114	0.74	−0.067	2.105	0.73	-	-	-	-
2-12	−0.033	2.048	0.56	−0.064	2.093	0.95	−0.052	2.060	0.83	-	-	-	-
2-13	0.013	1.953	0.58	−0.044	2.034	0.94	−0.146	2.154	0.97	-	-	-	-
2-14	−0.029	1.982	0.55	−0.028	1.954	0.49	−0.044	1.978	0.55	-	-	-	-
2-15	−0.046	2.048	0.94	−0.045	2.023	0.94	−0.163	2.157	0.94	-	-	-	-
2-16	0.005	1.973	0.04	−0.092	2.172	0.81	−0.209	2.374	0.93	-	-	-	-
2-17	−0.015	2.008	1.00	−0.033	2.025	0.99	−0.081	2.094	0.99	-	-	-	-
2-18	−0.010	2.008	1.00	−0.025	2.022	1.00	−0.059	2.067	1.00	-	-	-	-
2-19	−0.207	2.223	0.87	−0.226	2.210	0.91	−0.398	2.403	0.97	-	-	-	-
2-20	−0.027	2.039	0.91	−0.137	2.206	0.98	−0.155	2.039	0.97	-	-	-	-
2-21	−0.028	2.037	0.78	−0.185	2.313	0.97	−0.202	2.124	0.96	-	-	-	-
2-22	−0.073	2.104	0.95	−0.199	2.300	0.98	−0.289	2.180	0.98	-	-	-	-
2-23	−0.012	1.981	0.93	−0.083	2.075	0.89	−0.179	2.163	0.93	-	-	-	-
2-24	−0.071	2.089	0.93	−0.341	2.421	0.79	−1.623	3.841	0.98	-	-	-	-
2-25	−0.047	2.072	1.00	−0.153	2.211	0.97	−0.996	3.424	0.98	-	-	-	-
2-26	−0.081	2.124	0.97	−0.227	2.311	0.91	−0.640	2.875	0.93	-	-	-	-
2-27	0.014	1.966	0.28	−0.142	2.203	0.96	−0.445	2.623	0.99	-	-	-	-
2-28	−0.124	2.183	0.99	−0.310	2.462	0.99	−0.294	2.196	0.95	-	-	-	10,541.225
2-29	−0.118	2.161	0.95	−0.319	2.482	0.99	−0.218	2.044	0.91	-	-	-	11,702.275
2-30	−0.013	2.012	0.59	−0.041	2.059	0.68	−0.233	2.346	0.82	-	-	-	-
2-31	−0.023	2.032	0.91	−0.114	2.177	0.76	−0.360	2.532	0.86	-	-	-	-
2-32	−0.089	2.120	0.88	−0.205	2.290	0.79	−0.233	2.196	0.87	-	-	-	-
2-33	−0.051	2.052	0.85	−0.153	2.203	0.96	−0.244	2.277	0.93	-	-	-	-
2-34	−0.045	2.044	1.00	−0.122	2.137	0.96	−0.164	2.182	1.00	-	-	-	-
2-35	−0.028	2.007	0.93	−0.036	2.017	0.95	−0.090	2.052	0.96	-	-	-	-
2-36	−0.039	2.025	0.99	−0.044	2.027	0.98	−0.071	2.027	0.80	-	-	-	-
2-37	−0.018	2.006	0.95	−0.033	2.017	0.99	−0.126	2.088	0.97	-	-	-	-
2-38	−0.036	2.016	0.98	−0.052	2.033	0.99	−0.123	2.076	0.98	-	-	-	-
2-39	−0.070	2.078	0.89	−0.097	2.104	1.00	−0.210	2.234	0.96	-	-	-	-
2-40	−0.133	2.159	0.97	−0.184	2.206	0.90	−0.296	2.326	0.92	-	-	-	-
3-1	−0.149	2.074	0.99	−0.115	1.993	0.97	−0.189	2.069	0.91	-	-	-	-
3-2	0.026	1.897	0.66	−0.044	2.047	0.26	−0.092	2.153	0.92	-	-	-	-
3-3	−0.086	2.122	0.94	−0.132	2.141	0.94	−0.242	2.268	0.96	-0.270	2.294	0.975	-
3-4	−0.037	2.066	0.83	−0.046	2.078	0.94	−0.043	2.066	0.83	-	-	-	-
3-5	0.006	1.979	0.63	0.006	1.954	0.03	−0.035	2.037	0.18	-	-	-	-
3-6	−0.091	2.111	0.84	−0.126	2.153	0.74	−0.312	2.518	0.80	-	-	-	-
3-7	−0.033	2.040	0.74	−0.034	2.036	0.83	−0.026	1.999	0.50	-	-	-	-
3-8	−0.187	2.103	0.99	−0.227	2.118	0.98	−0.363	2.286	0.93	-	-	-	-
3-9	−0.082	2.002	0.88	−0.087	1.955	0.89	−0.150	2.017	0.91	-	-	-	-
3-10	−0.119	2.058	0.96	−0.122	1.978	0.98	−0.187	2.033	0.99	-	-	-	-
3-11	−0.358	2.297	0.99	−0.399	2.273	0.98	−0.560	2.445	0.94	-	-	-	-
3-12	−0.215	2.103	0.99	−0.218	2.036	0.94	−0.386	2.241	0.99	-	-	-	-
3-13	−0.253	2.143	0.97	−0.350	2.206	0.98	−0.382	2.188	0.98	-	-	-	-
3-14	−0.005	1.982	0.20	−0.165	2.249	0.91	−0.175	2.091	0.93	-	-	-	-
3-15	−0.026	2.022	0.81	−0.193	2.296	0.98	−0.193	2.121	0.97	-	-	-	-
3-16	−0.036	2.022	0.83	−0.133	2.119	0.94	−0.126	1.939	0.90	-	-	-	-
3-17	−0.109	2.160	0.86	−0.215	2.347	0.99	−0.382	2.553	0.93	-	-	-	-
3-18	−0.040	2.044	0.95	−0.075	2.069	0.85	−0.176	2.201	1.00	-	-	-	-

**Table A8:** Power function model parameter values based on the accelerated test data.

Group Number	*T*_1_			*T*_2_			*T*_3_			*T*_4_			*E_a_*/*R*
	*j*	*a*	*R* ^2^	*j*	*a*	*R* ^2^	*j*	*a*	*R* ^2^	*j*	*a*	*R* ^2^	
1-1	2.544 × 10^−2^	−0.763	0.72	2.415 × 10^−2^	−0.730	0.97	4.437 × 10^−2^	−0.792	0.93	-	-	-	-
1-2	2.598 × 10^−2^	−0.741	0.53	6.380 × 10^−3^	−0.411	0.90	4.883 × 10^−2^	−0.601	1.00	-	-	-	7838.00
1-3	2.007 × 10^−2^	−0.695	0.76	3.244 × 10^−2^	−0.822	0.88	1.975 × 10^−2^	−0.643	0.93	-	-	-	-
1-4	4.938 × 10^−3^	−0.369	0.93	3.250 × 10^−2^	−0.759	0.82	5.551 × 10^−2^	−0.630	0.99	-	-	-	
1-5	8.850 × 10^−26^	9.935	0.98	3.795 × 10^−3^	−0.634	1.00	1.414 × 10^−3^	−0.465	1.00	-	-	-	-
1-6	−6.380 × 10^−3^	−1.000	0.07	7.448 × 10^−96^	39.411	0.98	6.279 × 10^−4^	−0.163	0.98	-	-	-	-
1-7	2.197 × 10^−4^	−0.212	0.50	2.585 × 10^−4^	0.179	1.00	4.664 × 10^−4^	0.163	0.97	-	-	-	9018.60
1-8	1.064 × 10^−2^	−0.528	0.91	1.234 × 10^−2^	−0.444	0.92	1.912 × 10^−2^	−0.447	0.98	-	-	-	5941.50
1-9	1.394 × 10^−3^	−0.272	0.95	4.770 × 10^−2^	−0.803	0.71	3.935 × 10^−2^	−0.626	0.95	-	-	-	-
1-10	1.962	−0.997	0.55	4.718 × 10^−3^	−0.421	0.99	9.981 × 10^−2^	−0.799	1.00	-	-	-	-
1-11	1.619 × 10^−4^	−0.063	0.96	1.130 × 10^−6^	0.953	0.87	7.185 × 10^−2^	−0.785	0.98	-	-	-	-
1-12	2.385 × 10^−6^	0.543	0.29	3.827 × 10^−9^	1.733	0.95	1.743 × 10^−3^	−0.254	0.83	-	-	-	-
1-13	2.037 × 10^−9^	1.804	0.84	3.194 × 10^−6^	0.540	0.89	2.846 × 10^−3^	−0.356	0.87	-	-	-	-
1-14	1.013 × 10^−4^	−0.088	0.91	7.922 × 10^−4^	−0.122	0.98	7.698 × 10^−2^	−0.759	0.99	-	-	-	12,325.05
1-15	1.208 × 10^−3^	−0.488	0.44	1.275 × 10^−4^	0.392	0.97	3.236 × 10^−4^	0.286	1.00	-	-	-	-
1-16	4.008 × 10^−5^	0.411	0.95	1.829 × 10^−3^	−0.122	0.99	5.174 × 10^−3^	−0.278	1.00	-	-	-	2739.90
2-1	7.167 × 10^−3^	−0.282	0.95	1.200 × 10^−2^	−0.345	1.00	4.414 × 10^−2^	−0.549	1.00	-	-	-	3270.83
2-2	8.279 × 10^−6^	0.788	1.00	7.501 × 10^−4^	−0.030	0.93	3.867 × 10^−3^	−0.143	1.00	-	-	-	-
2-3	4.055 × 10^−4^	−0.052	0.97	9.899 × 10^−4^	−0.103	0.88	2.461 × 10^−2^	−0.662	0.96	6.861 × 10^−2^	−0.821	0.90	-
2-4	3.576 × 10^−4^	−0.031	0.97	1.077 × 10^−4^	0.279	1.00	1.424 × 10^−2^	−0.561	0.94	3.113 × 10^−2^	−0.682	0.99	-
2-5	1.541 × 10^−5^	0.831	1.00	1.224 × 10^−2^	−0.560	0.98	1.603 × 10^−2^	−0.434	0.99	-	-	-	-
2-6	1.680 × 10^−3^	−0.229	0.90	9.149 × 10^−4^	0.121	1.00	5.350 × 10^−5^	1.017	0.99	1.290 × 10^−2^	−0.111	0.99	12,985.25
2-7	2.825 × 10^−3^	−0.542	0.36	2.688 × 10^−3^	−0.297	1.00	6.515 × 10^−3^	−0.290	0.99	-	-	-	18,089.50
2-8	2.614 × 10^−2^	−0.802	0.94	1.844	−0.986	0.99	8.727 × 10^−2^	−0.901	0.82	-	-	-	-
2-9	1.531 × 10^−2^	−0.992	0.63	1.904	−0.992	0.96	2.610 × 10^−2^	−0.850	0.94	-	-	-	-
2-10	2.856 × 10^−3^	−0.469	0.90	2.545 × 10^−2^	−0.766	0.98	1.479 × 10^−2^	−0.604	0.93	-	-	-	-
2-11	3.023 × 10^−6^	0.746	1.00	7.183 × 10^−8^	1.573	1.00	1.202 × 10^−6^	1.054	0.98	-	-	-	-
2-12	9.671 × 10^−8^	1.422	0.92	3.581 × 10^−4^	−0.023	1.00	2.598 × 10^−3^	−0.379	0.97	-	-	-	-
2-13	1.948	−0.997	0.91	1.189 × 10^−2^	−0.654	0.99	2.176 × 10^−2^	−0.600	0.98	-	-	-	-
2-14	4.236 × 10^−2^	−0.855	0.95	6.825 × 10^−2^	−0.894	0.97	6.196 × 10^−2^	−0.858	0.95	-	-	-	-
2-15	2.315 × 10^−3^	−0.323	1.00	1.415 × 10^−2^	−0.638	1.00	1.248 × 10^−2^	−0.416	1.00	-	-	-	-
2-16	1.969	−0.999	0.69	1.052 × 10^−6^	0.971	0.98	1.680 × 10^−4^	0.217	1.00	-	-	-	-
2-17	4.827 × 10^−3^	−0.653	1.00	6.303 × 10^−3^	−0.577	1.00	3.285 × 10^−3^	−0.329	1.00	-	-	-	24,881.75
2-18	1.423 × 10^−3^	−0.537	0.99	3.115 × 10^−3^	−0.512	1.00	2.928 × 10^−3^	−0.362	1.00	-	-	-	22,290.25
2-19	9.004 × 10^−3^	−0.374	0.97	2.176 × 10^−2^	−0.508	0.97	3.256 × 10^−2^	−0.510	0.96	-	-	-	4094.98
2-20	5.975 × 10^−4^	−0.303	0.96	2.155 × 10^−3^	−0.261	1.00	1.071 × 10^−1^	−0.804	1.00	-	-	-	14,594.50
2-21	1.185 × 10^−3^	−0.392	0.92	1.836 × 10^−3^	−0.223	1.00	8.949 × 10^−2^	−0.760	0.99	-	-	-	14,118.75
2-22	1.721 × 10^−3^	−0.315	1.00	3.910 × 10^−3^	−0.309	0.97	1.259 × 10^−1^	−0.772	1.00	-	-	-	13,814.75
2-23	2.636 × 10^−2^	−0.867	1.00	6.991 × 10^−3^	−0.411	0.96	1.244 × 10^−2^	−0.376	1.00	-	-	-	33,783.00
2-24	1.300 × 10^−4^	0.378	1.00	2.918 × 10^−4^	0.557	0.98	2.012 × 10^−2^	−0.112	1.00	-	-	-	9013.75
2-25	7.034 × 10^−7^	1.306	0.90	1.649 × 10^−4^	0.430	1.00	3.788 × 10^−8^	2.889	0.99	-	-	-	4008.05
2-26	5.644 × 10^−7^	1.499	0.97	1.132 × 10^−3^	0.073	0.88	6.956 × 10^−4^	0.407	1.00	-	-	-	-
2-27	1.961	−0.996	0.62	3.364 × 10^−5^	0.750	1.00	3.885 × 10^−4^	0.449	0.99	-	-	-	-
2-28	3.004 × 10^−3^	−0.336	0.97	5.827 × 10^−3^	−0.306	0.99	1.237 × 10^−1^	−0.771	0.99	-	-	-	11,944.83
2-29	4.498 × 10^−3^	−0.391	1.00	5.092 × 10^−3^	−0.281	0.99	1.608 × 10^−1^	−0.826	0.99	-	-	-	-
2-30	1.262 × 10^−3^	−0.458	0.87	1.801 × 10^−5^	0.514	0.96	1.287 × 10^−4^	0.442	1.00	-	-	-	14,095.00
2-31	4.645 × 10^−4^	−0.255	0.84	2.652 × 10^−6^	1.050	1.00	3.231 × 10^−4^	0.343	1.00	-	-	-	11,212.75
2-32	1.040 × 10^−3^	−0.125	0.96	3.817 × 10^−4^	0.224	0.99	3.027 × 10^−2^	−0.518	0.99	-	-	-	7281.65
2-33	5.104 × 10^−3^	−0.485	0.96	2.799 × 10^−3^	−0.220	0.98	1.422 × 10^−2^	−0.421	0.99	-	-	-	8579.40
2-34	1.826 × 10^−3^	−0.240	1.00	1.075 × 10^−3^	0.081	1.00	2.339 × 10^−3^	−0.040	0.99	-	-	-	3772.58
2-35	1.057 × 10^−2^	−0.634	0.98	9.092 × 10^−3^	−0.580	0.96	1.578 × 10^−2^	−0.524	0.96	-	-	-	7594.93
2-36	6.211 × 10^−3^	−0.507	0.94	7.031 × 10^−3^	−0.505	0.96	1.665 × 10^−2^	−0.548	0.95	-	-	-	4509.43
2-37	6.748 × 10^−3^	−0.650	0.93	7.186 × 10^−3^	−0.557	0.97	1.660 × 10^−2^	−0.490	0.91	-	-	-	13,437.25
2-38	1.025 × 10^−2^	−0.604	0.95	7.448 × 10^−3^	−0.482	0.97	2.102 × 10^−2^	−0.533	0.94	-	-	-	7529.70
2-39	1.957 × 10^−3^	−0.211	0.99	5.042 × 10^−3^	−0.345	1.00	7.165 × 10^−3^	−0.271	1.00	-	-	-	3483.75
2-40	2.372 × 10^−3^	−0.138	1.00	5.258 × 10^−3^	−0.228	0.99	1.060 × 10^−2^	−0.286	0.99	-	-	-	2794.63
3-1	4.245 × 10^−2^	−0.640	0.99	8.477 × 10^−2^	−0.778	0.98	6.922 × 10^−2^	−0.684	0.97	-	-	-	-
3-2	1.887	−0.994	0.99	1.001 × 10^−2^	−0.668	0.65	1.718 × 10^−4^	0.119	0.97	-	-	-	-
3-3	1.400 × 10^−3^	−0.234	0.94	1.234 × 10^−2^	−0.504	0.99	2.088 × 10^−2^	−0.508	0.99	2.696 × 10^−2^	−0.538	0.98	5663.48
3-4	1.107 × 10^−5^	0.387	0.99	1.391 × 10^−4^	−0.005	1.00	3.947 × 10^−4^	−0.167	0.97	-	-	-	-
3-5	1.976	−0.999	0.94	1.949	−0.999	0.89	5.307 × 10^−3^	−0.593	0.77	-	-	-	-
3-6	1.537 × 10^−2^	−0.637	0.99	2.778 × 10^−2^	−0.687	0.97	1.685 × 10^−2^	−0.530	0.96	-	-	-	4242.43
3-7	2.750 × 10^−3^	−0.501	0.97	7.072 × 10^−3^	−0.643	0.98	2.373 × 10^−2^	−0.799	0.98	-	-	-	-
3-8	6.586 × 10^−2^	−0.709	0.98	7.619 × 10^−2^	−0.702	1.00	4.686 × 10^−2^	−0.542	1.00	-	-	-	-
3-9	6.432 × 10^−2^	−0.796	0.95	1.034 × 10^−1^	−0.837	0.98	9.157 × 10^−2^	−0.766	0.99	-	-	-	-
3-10	4.778 × 10^−2^	−0.716	0.97	1.101 × 10^−1^	−0.816	0.99	1.114 × 10^−1^	−0.777	1.00	-	-	-	-
3-11	5.738 × 10^−2^	−0.611	0.99	8.519 × 10^−2^	−0.644	0.99	5.731 × 10^−2^	−0.492	1.00	-	-	-	-
3-12	8.616 × 10^−2^	−0.733	0.98	1.266 × 10^−1^	−0.776	0.99	1.002 × 10^−1^	−0.679	0.99	-	-	-	-
3-13	8.678 × 10^−2^	−0.717	0.97	1.073 × 10^−1^	−0.711	0.99	1.276 × 10^−1^	−0.715	0.99	-	-	-	4194.55
3-14	2.204 × 10^−2^	−0.927	0.98	8.544 × 10^−3^	−0.481	0.96	8.924 × 10^−2^	−0.773	0.99	-	-	-	-
3-15	5.764 × 10^−3^	−0.633	0.95	2.588 × 10^−3^	−0.244	0.98	8.384 × 10^−2^	−0.756	0.99	-	-	-	16,538.75
3-16	1.317 × 10^−2^	−0.694	0.97	2.940 × 10^−2^	−0.649	0.99	1.618 × 10^−1^	−0.865	0.99	-	-	-	20,460.00
3-17	3.787 × 10^−3^	−0.382	0.97	2.465 × 10^−3^	−0.216	1.00	1.684 × 10^−2^	−0.447	0.99	-	-	-	4748.28
3-18	4.903 × 10^−3^	−0.559	0.96	1.289 × 10^−2^	−0.594	0.98	1.742 × 10^−2^	−0.537	1.00	-	-	-	7627.53

## Appendix C. Groups Meeting Old or New Criterion Based on Different Models

**Table A9:** Groups meeting old or new criterion based on different models.

New Group	Exponential		Modified Exponential		Single Logarithmic		Double Logarithmic		Power	
	Old	New	Old	New	Old	New	Old	New	Old	New
1-1	-	Y	-	-	-	Y	-	-	-	-
1-2	-	Y	-	Y	-	-	-	-	-	Y
1-3	-	-	-	-	-	-	-	-	-	-
1-4	-	Y	-	-	-	-	-	-	-	-
1-5	-	-	-	-	-	-	-	-	-	-
1-6	-	Y	-	-	-	Y	-	-	-	-
1-7	-	Y	-	Y	-	-	-	-	-	Y
1-8	-	Y	Y	Y	-	-	-	-	Y	Y
1-9	-	-	-	-	-	-	-	-	-	-
1-10	-	-	-	-	-	-	-	-	-	-
1-11	-	Y	-	-	-	Y	-	-	-	-
1-12	-	Y	-	-	-	-	-	-	-	-
1-13	-	Y	-	-	-	-	-	-	-	-
1-14	-	Y	Y	Y	Y	Y	-	-	Y	Y
1-15	-	Y	-	-	-	-	-	-	-	-
1-16	Y	Y	Y	Y	-	-	-	-	Y	Y
2-1	Y	Y	Y	Y	Y	Y	Y	Y	Y	Y
2-2	Y	Y	-	-	Y	Y	-	-	-	-
2-3	-	Y	-	-	-	-	-	-	-	-
2-4	-	Y	-	-	-	-	-	-	-	-
2-5	Y	Y	-	-	Y	Y	-	-	-	-
2-6	Y	-	Y	Y	-	-	-	-	Y	-
2-7	-	Y	-	Y	-	-	-	-	-	Y
2-8	-	Y	-	-	-	-	-	-	-	-
2-9	-	-	-	-	-	-	-	-	-	-
2-10	-	Y	-	-	-	-	-	-	-	-
2-11	Y	Y	-	-	-	-	-	-	-	-
2-12	Y	Y	-	-	-	-	-	-	-	-
2-13	-	Y	-	-	-	-	-	-	-	-
2-14	-	-	-	-	-	-	-	-	-	-
2-15	Y	Y	-	-	-	-	-	-	-	-
2-16	-	Y	-	-	-	-	-	-	-	-
2-17	-	Y	Y	Y	-	-	-	-	Y	Y
2-18	Y	Y	Y	Y	-	-	-	-	Y	Y
2-19	Y	Y	Y	Y	Y	Y	-	-	Y	Y
2-20	-	Y	Y	Y	Y	-	-	-	Y	Y
2-21	-	Y	Y	Y	-	-	-	-	Y	Y
2-22	-	Y	Y	Y	Y	Y	-	-	Y	Y
2-23	-	Y	Y	Y	-	-	-	-	Y	Y
2-24	Y	Y	Y	Y	Y	-	-	-	Y	Y
2-25	-	Y	Y	Y	-	-	-	-	Y	Y
2-26	-	Y	-	-	-	-	-	-	-	-
2-27	-	Y	-	-	-	-	-	-	-	-
2-28	-	Y	Y	Y	Y	Y	Y	Y	Y	Y
2-29	-	-	Y	Y	Y	Y	Y	Y	-	-
2-30	-	Y	Y	Y	-	-	-	-	Y	Y
2-31	Y	Y	Y	Y	-	-	-	-	Y	Y
2-32	Y	Y	Y	Y	Y	Y	-	-	Y	Y
2-33	-	Y	Y	Y	-	-	-	-	Y	Y
2-34	Y	Y	Y	Y	-	-	-	-	Y	Y
2-35	-	Y	Y	Y	-	-	-	-	Y	Y
2-36	-	Y	Y	Y	-	-	-	-	Y	Y
2-37	-	Y	Y	Y	-	-	-	-	Y	Y
2-38	-	Y	Y	Y	-	-	-	-	Y	Y
2-39	Y	Y	Y	Y	-	-	-	-	Y	Y
2-40	Y	Y	Y	Y	Y	Y	-	-	Y	Y
3-1	-	-	-	-	-	-	-	-	-	-
3-2	-	Y	-	-	-	-	-	-	-	-
3-3	Y	Y	Y	Y	Y	Y	-	-	Y	-
3-4	Y	Y	-	-	-	-	-	-	-	-
3-5	-	Y	-	-	-	-	-	-	-	-
3-6	-	Y	Y	Y	-	-	-	-	Y	Y
3-7	-	Y	-	-	-	-	-	-	-	-
3-8	-	-	-	-	-	-	-	-	-	-
3-9	-	-	-	-	-	-	-	-	-	-
3-10	-	-	-	-	-	-	-	-	-	-
3-11	-	-	-	-	-	-	-	-	-	-
3-12	-	-	-	-	-	-	-	-	-	-
3-13	-	-	Y	Y	Y	Y	-	-	Y	Y
3-14	-	-	-	-	-	-	-	-	-	-
3-15	-	Y	-	-	-	-	-	-	-	-
3-16	-	-	Y	Y	Y	Y	-	-	Y	Y
3-17	Y	Y	Y	Y	-	-	-	-	Y	Y
3-18	-	Y	Y	Y	-	-	-	-	Y	Y

Note: ‘Y’ denotes the group meets the criteria, ‘-’ denotes the group does not meet the criterion. ‘Old’ denotes the old criterion, ‘New’ denotes the new criterion.

## Appendix D. Different Model Parameters Values When Bar Is Exposed to 5–30 °C

**Table A10:** Exponential model parameter value-based transformed data.

Group Number	5C	10C	15C	20C	25C	30C
	τ	τ	τ	τ	τ	τ
1-1	779.96	735.80	695.54	658.74	625.03	594.08
1-2	1290.15	1065.12	885.21	740.35	622.91	527.11
1-4	1197.19	994.74	831.85	699.90	592.30	504.00
1-6	1.407 × 10^8^	3.352 × 10^7^	8.396 × 10^6^	2.205 × 10^6^	6.054 × 10^5^	1.735 × 10^5^
1-7	20,533.51	13,708.07	9280.68	6367.41	4424.18	3111.13
1-8	2346.69	1790.32	1378.74	1071.29	839.47	663.12
1-11	17,974.55	12,102.71	8261.69	5713.62	4000.60	2834.30
1-12	38,018.79	26,216.58	18,312.86	12,949.44	9263.89	6700.94
1-13	27,191.06	19,676.09	14,398.82	10,649.79	7956.99	6002.50
1-14	49,049.03	28,480.50	16,852.25	10,151.77	6220.26	3873.41
1-15	9890.24	7008.91	5026.72	3646.23	2673.50	1980.43
1-16	5738.58	4127.61	3003.01	2208.68	1641.25	1231.63
2-1	342.02	306.66	276.00	249.29	225.94	205.44
2-2	4117.38	3029.35	2252.69	1692.17	1283.38	982.26
2-3	1954.39	1744.86	1563.95	1407.03	1270.37	1150.83
2-4	2147.91	1905.21	1696.97	1517.48	1362.07	1226.94
2-5	5671.37	3724.16	2481.46	1676.49	1147.64	795.49
2-6	30,535.13	14,658.92	7218.79	3641.84	1879.94	991.83
2-7	17,805.61	10,692.83	6536.01	4062.82	2566.05	1645.46
2-8	1757.65	1353.06	1051.11	823.61	650.65	518.00
2-10	3060.04	2589.54	2204.13	1886.43	1622.97	1403.26
2-11	4571.68	4073.46	3644.08	3272.44	2949.26	2667.17
2-12	4397.33	3826.49	3345.86	2939.05	2592.95	2297.05
2-13	14,843.14	10,264.39	7189.51	5097.33	3655.89	2650.98
2-15	1940.18	1539.51	1231.42	992.52	805.77	658.67
2-16	14,668.65	10,860.38	8125.14	6139.25	4682.53	3603.51
2-17	63,478.95	41,681.09	27,770.81	18,760.89	12,841.97	8901.03
2-18	376,461.42	214,830.99	125,005.21	74,093.73	44,694.39	27,413.66
2-19	839.80	693.55	576.58	482.38	405.98	343.64
2-20	21,447.30	13,401.02	8511.19	5489.95	3593.62	2385.44
2-21	16,894.13	10,753.55	6953.06	4563.10	3037.24	2048.94
2-22	7309.22	4866.02	3285.56	2248.35	1558.27	1093.14
2-23	1987.55	1503.95	1149.08	886.04	689.20	540.56
2-24	11,390.96	5680.03	2901.54	1516.55	810.10	441.77
2-25	15,376.97	8500.29	4796.57	2759.98	1617.82	965.18
2-26	5079.47	3256.46	2120.20	1400.76	938.40	637.02
2-27	17,168.08	10,053.04	5997.07	3641.11	2248.01	1410.16
2-28	3034.10	2195.98	1607.31	1189.03	888.54	670.40
2-30	34,244.01	21,492.26	13,708.84	8879.34	5835.62	3888.72
2-31	53,540.76	30,292.19	17,480.78	10,278.65	6152.43	3745.49
2-32	6149.21	4101.54	2774.45	1901.95	1320.46	927.84
2-33	4495.55	3266.56	2400.00	1781.97	1336.36	1011.74
2-34	1068.16	903.80	769.19	658.24	566.25	489.54
2-35	1576.05	1324.51	1119.87	952.27	814.17	699.71
2-36	1464.54	1244.45	1063.43	913.62	788.93	684.57
2-37	4500.66	3287.62	2427.83	1811.54	1365.03	1038.22
2-38	1612.29	1308.71	1070.02	880.88	729.93	608.60
2-39	1212.72	1005.19	838.62	704.00	594.46	504.78
2-40	632.97	538.72	461.08	396.73	343.09	298.11
3-2	2250.36	2135.25	2029.70	1932.73	1843.42	1760.97
3-3	2463.25	1907.53	1490.34	1174.24	932.61	746.36
3-4	6985.35	6311.62	5723.00	5206.61	4751.83	4349.92
3-5	61,958.44	44,309.96	32,059.39	23,453.28	17,338.23	12,945.96
3-6	1860.13	1636.60	1446.35	1283.61	1143.75	1023.01
3-7	3540.53	3230.24	2956.54	2714.23	2498.94	2306.98
3-15	10,251.68	6891.59	4697.10	3243.54	2267.78	1604.40
3-17	1648.09	1349.28	1112.33	923.05	770.79	647.48
3-18	3982.83	3131.31	2482.49	1983.75	1597.18	1295.17

**Table A11:** Modified exponential model parameter values-based transformed data.

Group Number	5C	5C	10C	10C	15C	15C	20C	20C	25C	25C	30C	30C
	n	τ	n	τ	n	τ	n	τ	n	τ	n	τ
1-2	0.396	94.81	0.396	77.70	0.396	64.12	0.396	53.26	0.396	44.52	0.396	37.43
1-7	0.553	1518.69	0.553	1098.58	0.553	803.68	0.553	594.23	0.553	443.85	0.553	334.74
1-8	0.603	225.11	0.603	178.42	0.603	142.56	0.603	114.78	0.603	93.09	0.603	76.02
1-14	0.516	1064.92	0.516	709.22	0.516	479.04	0.516	327.92	0.516	227.35	0.516	159.54
1-16	1.084	2605.86	1.084	2147.54	1.084	1781.77	1.084	1487.85	1.084	1249.73	1.084	1055.79
2-1	0.647	67.84	0.647	60.17	0.647	53.58	0.647	47.90	0.647	42.99	0.647	38.72
2-6	1.182	4.913 × 10^5^	1.182	1.823 × 10^5^	1.182	7.001 × 10^4^	1.182	2.778 × 10^4^	1.182	1.137 × 10^4^	1.182	4.793 × 10^3^
2-7	0.421	1742.16	0.421	1058.75	0.421	654.65	0.421	411.47	0.421	262.69	0.421	170.21
2-17	0.288	3774.06	0.288	2357.03	0.288	1496.29	0.288	964.71	0.288	631.21	0.288	418.82
2-18	0.406	30,790.07	0.406	17,060.45	0.406	9648.70	0.406	5564.05	0.406	3268.37	0.406	1953.86
2-19	0.609	88.42	0.609	75.96	0.609	65.60	0.609	56.94	0.609	49.65	0.609	43.49
2-20	0.417	455.11	0.417	309.41	0.417	213.20	0.417	148.78	0.417	105.08	0.417	75.08
2-21	0.421	406.76	0.421	278.73	0.421	193.52	0.421	136.04	0.421	96.77	0.421	69.62
2-22	0.449	319.46	0.449	219.61	0.449	152.94	0.449	107.84	0.449	76.93	0.449	55.49
2-23	0.138	77.09	0.138	56.70	0.138	42.15	0.138	31.65	0.138	24.00	0.138	18.36
2-24	1.486	1.893 × 10^5^	1.486	8.305 × 10^4^	1.486	3.749 × 10^4^	1.486	1.739 × 10^4^	1.486	8.276 × 10^3^	1.486	4.037 × 10^3^
2-25	2.354	7.321 × 10^6^	2.354	3.836 × 10^6^	2.354	2.056 × 10^6^	2.354	1.125 × 10^6^	2.354	6.288 × 10^5^	2.354	3.579 × 10^5^
2-28	0.400	101.25	0.400	76.24	0.400	57.98	0.400	44.50	0.400	34.47	0.400	26.92
2-29	0.317	59.29	0.317	45.34	0.317	35.00	0.317	27.26	0.317	21.41	0.317	16.94
2-30	1.328	1.991 × 10^8^	1.328	5.802 × 10^7^	1.328	1.765 × 10^7^	1.328	5.590 × 10^6^	1.328	1.840 × 10^6^	1.328	6.284 × 10^5^
2-31	1.194	2.346 × 10^6^	1.194	9.721 × 10^5^	1.194	4.152 × 10^5^	1.194	1.826 × 10^5^	1.194	8.253 × 10^4^	1.194	3.829 × 10^4^
2-32	0.886	4037.84	0.886	2663.12	0.886	1781.97	0.886	1208.84	0.886	830.77	0.886	578.07
2-33	0.604	827.08	0.604	588.50	0.604	423.72	0.604	308.52	0.604	227.04	0.604	168.78
2-34	0.731	424.03	0.731	353.53	0.731	296.68	0.731	250.44	0.731	212.59	0.731	181.48
2-35	0.423	178.74	0.423	144.44	0.423	117.59	0.423	96.41	0.423	79.57	0.423	66.09
2-36	0.527	174.83	0.527	149.60	0.527	128.69	0.527	111.28	0.527	96.70	0.527	84.41
2-37	0.441	813.97	0.441	550.34	0.441	377.19	0.441	261.87	0.441	184.04	0.441	130.86
2-38	0.474	207.66	0.474	164.06	0.474	130.68	0.474	104.90	0.474	84.83	0.474	69.08
2-39	0.829	598.83	0.829	494.95	0.829	411.80	0.829	344.80	0.829	290.41	0.829	246.00
2-40	0.851	321.93	0.851	275.65	0.851	237.25	0.851	205.28	0.851	178.48	0.851	155.89
3-3	0.607	245.02	0.607	196.38	0.607	158.61	0.607	129.03	0.607	105.71	0.607	87.17
3-6	0.455	76.92	0.455	67.94	0.455	60.27	0.455	53.69	0.455	48.01	0.455	43.08
3-13	0.375	9.84	0.375	9.01	0.375	8.28	0.375	7.63	0.375	7.05	0.375	6.53
3-16	0.214	48.15	0.214	36.61	0.214	28.10	0.214	21.76	0.214	17.00	0.214	13.39
3-17	0.711	438.32	0.711	352.96	0.711	286.38	0.711	234.01	0.711	192.53	0.711	159.42
3-18	0.455	207.28	0.455	164.98	0.455	132.35	0.455	106.98	0.455	87.09	0.455	71.38

**Table A12:** Single logarithmic model parameter values-based transformed data.

Group Number	0−110	5C	5C	10C	10C	15C	15C	20C	20C	25C	25C	30C	30C
		a	b	a	b	a	b	a	b	a	b	a	b
1-1	Y	−7.05	103.68	−7.05	102.74	−7.05	101.84	−7.05	100.97	−7.05	100.12	−7.05	99.31
1-6	Y	−4.96	119.22	−4.96	117.22	−4.96	115.28	−4.96	113.41	−4.96	111.60	−4.96	109.85
1-11	-	−9.17	141.94	−9.17	135.95	−9.17	130.16	−9.17	124.57	−9.17	119.16	−9.17	113.94
1-14	-	−5.85	139.41	−5.85	131.98	−5.85	124.81	−5.85	117.88	−5.85	111.19	−5.85	104.72
2-1	-	−52.93	182.72	−52.94	178.96	−52.94	175.34	−52.94	171.84	−52.94	168.46	−52.93	165.18
2-2	Y	−19.33	147.37	−19.33	143.16	−19.33	139.10	−19.33	135.18	−19.33	131.38	−19.33	127.72
2-5	Y	−8.01	127.51	−8.01	123.03	−8.01	118.71	−8.01	114.54	−8.01	110.50	−8.01	106.60
2-19	-	−38.89	158.26	−38.88	154.63	−38.88	151.13	−38.89	147.76	−38.88	144.49	−38.89	141.34
2-20	-	−7.79	142.44	−7.79	134.66	−7.79	127.16	−7.79	119.90	−7.79	112.89	−7.79	106.12
2-22	-	−19.71	166.92	−19.71	157.88	−19.71	149.16	−19.71	140.74	−19.71	132.60	−19.71	124.73
2-24	-	−30.13	206.55	−30.13	191.65	−30.13	177.26	−30.13	163.37	−30.13	149.94	−30.13	136.96
2-28	Y	−25.78	168.32	−25.78	160.25	−25.78	152.47	−25.78	144.95	−25.78	137.68	−25.78	130.65
2-29	Y	−21.39	156.03	−21.39	148.33	−21.39	140.90	−21.39	133.73	−21.39	126.80	−21.39	120.09
2-32	-	−20.61	163.58	−20.61	156.77	−20.61	150.18	−20.61	143.83	−20.61	137.68	−20.61	131.74
2-40	Y	−29.01	149.22	−29.01	145.60	−29.01	142.10	−29.01	138.72	−29.01	135.46	−29.01	132.30
3-3	Y	−22.78	154.51	−22.78	148.76	−22.78	143.22	−22.78	137.87	−22.78	132.69	−22.78	127.69
3-13	-	−30.22	115.68	−30.22	112.76	−30.22	109.95	−30.22	107.23	−30.22	104.60	−30.22	102.06
3-16	Y	−9.42	133.39	−9.42	126.26	−9.42	119.38	−9.42	112.74	−9.42	106.31	−9.42	100.11

Note: ‘Y’ denotes predicted strength retention vary from 0 to 110%. ‘-’ denotes predicted strength retention smaller than 0, or larger than 110%, or shows fluctuation with the increase of exposure time.

**Table A13:** Double logarithmic model parameter values-based transformed data.

Group Number	0−110	5C	5C	10C	10C	15C	15C	20C	20C	25C	25C	30C	30C
		a	b	a	b	a	b	a	b	a	b	a	b
2-1	Y	−0.513	2.906	−0.513	2.879	−0.513	2.853	−0.513	2.828	−0.513	2.804	−0.513	2.781
2-28	Y	−0.185	2.508	−0.185	2.454	−0.185	2.402	−0.185	2.352	−0.185	2.304	−0.185	2.257
2-29	Y	−0.162	2.446	−0.162	2.394	−0.162	2.344	−0.162	2.295	−0.162	2.248	−0.162	2.202

Note: ‘Y’ denotes predicted strength retention which vary from 0 to 110%. ‘-’ denotes predicted strength retention smaller than 0, or larger than 110%, or shows fluctuation with the increase in exposure time.

**Table A14:** Power model parameter values-based transformed data.

Group Number	0–110	5C	5C	10C	10C	15C	15C	20C	20C	25C	25C	30C	30C
		j	a	j	a	j	a	j	a	j	a	j	a
1-2	Y	6.432 × 10^−3^	−0.636	7.708 × 10^−3^	−0.636	9.180 × 10^−3^	−0.636	1.087 × 10^−2^	−0.636	1.279 × 10^−2^	−0.636	1.498 × 10^−2^	−0.636
1-7	Y	3.350 × 10^−4^	−0.445	4.602 × 10^−4^	−0.445	6.252 × 10^−4^	−0.445	8.406 × 10^−4^	−0.445	1.119 × 10^−3^	−0.445	1.476 × 10^−3^	−0.445
1-8	-	3.740 × 10^−3^	−0.478	4.554 × 10^−3^	−0.478	5.506 × 10^−3^	−0.478	6.615 × 10^−3^	−0.478	7.899 × 10^−3^	−0.478	9.377 × 10^−3^	−0.478
1-14	Y	7.582 × 10^−4^	−0.534	1.092 × 10^−3^	−0.534	1.553 × 10^−3^	−0.534	2.183 × 10^−3^	−0.534	3.032 × 10^−3^	−0.534	4.167 × 10^−3^	−0.534
1-16	-	2.278 × 10^−4^	0.051	2.736 × 10^−4^	0.051	3.264 × 10^−4^	0.051	3.870 × 10^−4^	0.051	4.565 × 10^−4^	0.051	5.353 × 10^−4^	0.051
2-1	-	1.454 × 10^−2^	−0.510	1.609 × 10^−2^	−0.510	1.776 × 10^−2^	−0.510	1.953 × 10^−2^	−0.510	2.140 × 10^−2^	−0.510	2.339 × 10^−2^	−0.510
2-6	-	5.381 × 10^−6^	0.020	1.248 × 10^−5^	0.020	2.810 × 10^−5^	0.020	6.154 × 10^−5^	0.020	1.313 × 10^−4^	0.020	2.732 × 10^−4^	0.020
2-7	Y	3.092 × 10^−4^	−0.579	5.014 × 10^−4^	−0.579	7.995 × 10^−4^	−0.579	1.255 × 10^−3^	−0.579	1.940 × 10^−3^	−0.579	2.956 × 10^−3^	−0.579
2-17	Y	1.412 × 10^−4^	−0.709	2.237 × 10^−4^	−0.709	3.488 × 10^−4^	−0.709	5.356 × 10^−4^	−0.709	8.107 × 10^−4^	−0.709	1.210 × 10^−3^	−0.709
2-18	Y	1.794 × 10^−5^	−0.592	3.195 × 10^−5^	−0.592	5.578 × 10^−5^	−0.592	9.557 × 10^−5^	−0.592	1.608 × 10^−4^	−0.592	2.659 × 10^−4^	−0.592
2-19	-	9.060 × 10^−3^	−0.495	1.033 × 10^−2^	−0.495	1.172 × 10^−2^	−0.495	1.325 × 10^−2^	−0.495	1.491 × 10^−2^	−0.495	1.672 × 10^−2^	−0.495
2-20	Y	1.711 × 10^−3^	−0.629	2.414 × 10^−3^	−0.629	3.365 × 10^−3^	−0.629	4.638 × 10^−3^	−0.629	6.324 × 10^−3^	−0.629	8.534 × 10^−3^	−0.629
2-21	Y	1.960 × 10^−3^	−0.627	2.738 × 10^−3^	−0.627	3.780 × 10^−3^	−0.627	5.161 × 10^−3^	−0.627	6.975 × 10^−3^	−0.627	9.332 × 10^−3^	−0.627
2-22	Y	3.076 × 10^−3^	−0.635	4.238 × 10^−3^	−0.635	5.773 × 10^−3^	−0.635	7.783 × 10^−3^	−0.635	1.039 × 10^−2^	−0.635	1.373 × 10^−2^	−0.635
2-23	Y	6.714 × 10^−3^	−0.862	9.016 × 10^−3^	−0.862	1.199 × 10^−2^	−0.862	1.578 × 10^−2^	−0.862	2.058 × 10^−2^	−0.862	2.661 × 10^−2^	−0.862
2-24	-	2.266 × 10^−5^	0.169	4.425 × 10^−5^	0.169	8.439× 10^−5^	0.169	1.575 × 10^−4^	0.169	2.877 × 10^−4^	0.169	5.154 × 10^−4^	0.169
2-25	-	2.119 × 10^−12^	3.076	5.976 × 10^−12^	3.076	1.626 × 10^−11^	3.076	4.282 × 10^−11^	3.076	1.088 × 10^−10^	3.076	2.688 × 10^−10^	3.076
2-28	Y	8.937 × 10^−3^	−0.686	1.134 × 10^−2^	−0.686	1.427 × 10^−2^	−0.686	1.782 × 10^−2^	−0.686	2.208 × 10^−2^	−0.686	2.717 × 10^−2^	−0.686
2-30	Y	1.156 × 10^−8^	0.238	3.498 × 10^−8^	0.238	1.019 × 10^−7^	0.238	2.861 × 10^−7^	0.238	7.759 × 10^−7^	0.238	2.037 × 10^−6^	0.238
2-31	-	7.002 × 10^−7^	0.104	1.536 × 10^−6^	0.104	3.279 × 10^−6^	0.104	6.820 × 10^−6^	0.104	1.384 × 10^−5^	0.104	2.744 × 10^−5^	0.104
2-32	-	1.930 × 10^−4^	−0.174	2.828 × 10^−4^	−0.174	4.088 × 10^−4^	−0.174	5.836 × 10^−4^	−0.174	8.232 × 10^−4^	−0.174	1.148 × 10^−3^	−0.174
2-33	-	8.359 × 10^−4^	−0.432	1.139 × 10^−3^	−0.432	1.536 × 10^−3^	−0.432	2.050 × 10^−3^	−0.432	2.710 × 10^−3^	−0.432	3.549 × 10^−3^	−0.432
2-34	-	1.247 × 10^−3^	−0.277	1.483 × 10^−3^	−0.277	1.753 × 10^−3^	−0.277	2.060 × 10^−3^	−0.277	2.407 × 10^−3^	−0.277	2.799 × 10^−3^	−0.277
2-35	Y	3.031 × 10^−3^	−0.583	3.705 × 10^−3^	−0.583	4.498 × 10^−3^	−0.583	5.424 × 10^−3^	−0.583	6.500 × 10^−3^	−0.583	7.743 × 10^−3^	−0.583
2-36	-	3.095 × 10^−3^	−0.487	3.585 × 10^−3^	−0.487	4.131 × 10^−3^	−0.487	4.738 × 10^−3^	−0.487	5.408 × 10^−3^	−0.487	6.147 × 10^−3^	−0.487
2-37	Y	7.488 × 10^−4^	−0.570	1.081 × 10^−3^	−0.570	1.540 × 10^−3^	−0.570	2.168 × 10^−3^	−0.570	3.018 × 10^−3^	−0.570	4.155 × 10^−3^	−0.570
2-38	Y	2.760 × 10^−3^	−0.542	3.436 × 10^−3^	−0.542	4.245 × 10^−3^	−0.542	5.207 × 10^−3^	−0.542	6.343 × 10^−3^	−0.542	7.677 × 10^−3^	−0.542
2-39	-	9.731 × 10^−4^	−0.199	1.162 × 10^−3^	−0.199	1.379 × 10^−3^	−0.199	1.626 × 10^−3^	−0.199	1.908 × 10^−3^	−0.199	2.227 × 10^−3^	−0.199
2-40	-	1.999 × 10^−3^	−0.206	2.301 × 10^−3^	−0.206	2.636 × 10^−3^	−0.206	3.006 × 10^−3^	−0.206	3.413 × 10^−3^	−0.206	3.858 × 10^−3^	−0.206
3-3	-	3.075 × 10^−3^	−0.459	3.734 × 10^−3^	−0.459	4.505 × 10^−3^	−0.459	5.400 × 10^−3^	−0.459	6.433 × 10^−3^	−0.459	7.620 × 10^−3^	−0.459
3-6	Y	8.066 × 10^−3^	−0.586	9.017 × 10^−3^	−0.586	1.004 × 10^−2^	−0.586	1.114 × 10^−2^	−0.586	1.232 × 10^−2^	−0.586	1.357 × 10^−2^	−0.586
3-13	-	6.292 × 10^−2^	−0.712	6.794 × 10^−2^	−0.712	7.316 × 10^−2^	−0.712	7.858 × 10^−2^	−0.712	8.421 × 10^−2^	−0.712	9.004 × 10^−2^	−0.712
3-16	Y	1.337 × 10^−2^	−0.814	1.702 × 10^−2^	−0.814	2.148 × 10^−2^	−0.814	2.690 × 10^−2^	−0.814	3.344 × 10^−2^	−0.814	4.127 × 10^−2^	−0.814
3-17	-	1.629 × 10^−3^	−0.351	1.981 × 10^−3^	−0.351	2.393 × 10^−3^	−0.351	2.871 × 10^−3^	−0.351	3.425 × 10^−3^	−0.351	4.061 × 10^−3^	−0.351
3-18	Y	2.841 × 10^−3^	−0.565	3.506 × 10^−3^	−0.565	4.296 × 10^−3^	−0.565	5.228 × 10^−3^	−0.565	6.320 × 10^−3^	−0.565	7.592 × 10^−3^	−0.565

Note: ‘Y’ denotes predicted strength retention which vary from 0 to 110%. ‘-’ denotes predicted strength retention smaller than 0, or larger than 110%, or shows fluctuation with the increase in exposure time.
